# Overview of Monitoring, Diagnostics, Aging Analysis, and Maintenance Strategies in High-Voltage AC/DC XLPE Cable Systems

**DOI:** 10.3390/s25227096

**Published:** 2025-11-20

**Authors:** Kazem Emdadi, Majid Gandomkar, Ali Aranizadeh, Behrooz Vahidi, Mirpouya Mirmozaffari

**Affiliations:** 1 Department of Electrical Engineering, Sav. C., Islamic Azad University, Saveh 39197-15179, Iran; emdadi_kazem@stu.iau-saveh.ac.ir (K.E.); majid.gandomkar@iau.ac.ir (M.G.); 2 Department of Electrical Engineering, Amirkabir University of Technology (Tehran Polytechnic), Tehran 3715879817, Iran; aliarani@aut.ac.ir; 3 Department of Industrial Engineering, Dalhousie University, 5269 Morris Street, Halifax, NS B3H 4R2, Canada

**Keywords:** high-voltage cable systems, partial discharge detection, XLPE insulation aging, HVDC diagnostics, machine learning in fault analysis, condition-based maintenance

## Abstract

High-voltage (HV) cable systems—particularly those insulated with cross-linked polyethylene (XLPE)—are increasingly deployed in both AC and DC applications due to their excellent electrical and mechanical performance. However, their long-term reliability is challenged by partial discharges (PD), insulation aging, space charge accumulation, and thermal and electrical stresses. This review provides a comprehensive survey of the state-of-the-art technologies and methodologies across several domains critical to the assessment and enhancement of cable reliability. It covers advanced condition monitoring (CM) techniques, including sensor-based PD detection, signal acquisition, and denoising methods. Aging mechanisms under various stressors and lifetime estimation approaches are analyzed, along with fault detection and localization strategies using time-domain, frequency-domain, and hybrid methods. Physics-based and data-driven models for PD behavior and space charge dynamics are discussed, particularly under DC conditions. The article also reviews the application of numerical tools such as FEM for thermal and field stress analysis. A dedicated focus is given to machine learning (ML) and deep learning (DL) models for fault classification and predictive maintenance. Furthermore, standards, testing protocols, and practical issues in sensor deployment and calibration are summarized. The review concludes by evaluating intelligent maintenance approaches—including condition-based and predictive strategies—framed within real-world asset management contexts. The paper aims to bridge theoretical developments with field-level implementation challenges, offering a roadmap for future research and practical deployment in resilient and smart power grids. This review highlights a clear gap in fully integrated AC/DC diagnostic and aging analyses for XLPE cables. We emphasize the need for unified physics-based and ML-driven frameworks to address HVDC space-charge effects and multi-stress degradation. These insights provide concise guidance for advancing reliable and scalable cable assessment.

## 1. Introduction

High-voltage (HV) and medium-voltage (MV) cable systems constitute the backbone of modern power transmission and distribution networks. These cables are increasingly deployed in dense urban areas, industrial zones, offshore installations, and renewable energy infrastructures due to their superior reliability, aesthetic appeal, and lower land-use requirements compared to overhead lines. The primary insulating material in these cables—cross-linked polyethylene (XLPE)—exhibits excellent dielectric, mechanical, and thermal properties, making it the de facto standard for both HVAC and HVDC applications.

Despite these advantages, the long-term reliability of power cables is challenged by several operational stressors. These include thermal cycling, electrical over-stress, moisture ingress, mechanical strain, and environmental aging. Over time, such stressors degrade the insulation system, leading to phenomena such as partial discharges (PD), electrical treeing, and space charge accumulation. The consequences of insulation failure are often severe, resulting in prolonged outages, safety hazards, and high repair costs—especially for underground and submarine installations where fault location and repair are difficult.

Traditional maintenance practices, which rely on time-based or event-driven interventions, are increasingly seen as inadequate in the context of aging infrastructure and growing demands for grid reliability. In response, utilities and asset managers are shifting toward condition-based maintenance (CBM) and predictive diagnostics, supported by real-time monitoring technologies, advanced signal processing, and intelligent fault classification systems. These shifts reflect the broader movement toward digital asset management and smart grid transformation.

This review comprehensively surveys the recent advancements in the diagnostics, modeling, and reliability assessment of high-voltage power cables. It begins by examining condition monitoring methodologies, with a particular focus on PD detection techniques, sensor technologies, and advanced signal acquisition methods that are critical for real-time assessment and early fault detection [1,2,3,4,5,6,7,8,9,10,11,12,13,14,15,16,17,18,19,20,21,22,23,24,25,26,27,28,29,30,31,32,33,34,35,36]. The mechanisms of insulation aging and degradation under electrical, thermal, and environmental stressors are then discussed, especially in the context of XLPE-insulated systems, alongside various lifetime estimation models [37,38,39,40,41,42,43,44,45,46,47,48,49,50,51,52,53,54,55,56].

Further, the article reviews fault detection and localization techniques, comparing time-domain, frequency-domain, and hybrid diagnostic approaches for accurately pinpointing fault origins in cable systems [4,5,8,9,15,17,22,24,27,28,57,58,59,60,61,62,63,64]. In-depth analyses are also provided on the modeling and simulation of PD phenomena, incorporating both physics-based and data-driven models to interpret discharge characteristics under diverse stress conditions [1,3,6,10,11,13,14,15,16,17,18,19,21,23,25,31,43,59,60,61,62,65,66,67,68,69,70,71,72,73,74,75,76,77,78,79,80,81,82,83,84,85,86,87,88,89,90,91].

The dielectric behavior of cables under both AC and DC operations is addressed, highlighting phenomena such as space charge accumulation, polarity reversal, thermal stress, and environmental aging effects [38,41,42,43,47,48,50,53,54,55,65,66,69,82,84,88,91,92,93,94,95,96,97,98,99,100,101]. Numerical simulation tools, especially FEM-based electric field and thermal stress models, are then evaluated for their role in understanding cable component performance and failure initiation mechanisms [37,68,75,84,86,92,93,95,98,99,102]. According to [103], data-driven fault diagnosis has become essential for enhancing the safety and reliability of Railway Point Machines through continuous sensor-based monitoring.

The review also investigates recent applications of machine learning and deep learning for PD pattern classification, fault prediction, and intelligent condition assessment [8,14,15,16,17,19,22,26,27,28,34,58,59,61,62,63,64,70,104]. Specific challenges and design considerations for HVDC cable systems are explored, including life estimation under bipolar stress, insulation structure optimization, and failure mechanisms unique to DC operation [39,40,51,52,56,84,88,93,95,100,101,105,106,107,108,109,110,111,112,113,114,115,116,117,118,119,120,121].

Additionally, the paper summarizes existing testing protocols, measurement techniques, and evolving standardization practices, focusing on issues such as calibration, measurement uncertainty, and interoperability of diagnostic tools [1,3,6,10,11,12,13,17,18,20,21,23,31,57,58,59,60,72,73,74,80,85,87,90,100,118,122,123]. Finally, various asset management strategies are discussed, including condition-based and predictive maintenance, optimization under uncertainty, and reliability-centered planning methods that enhance operational efficiency and system resilience [22,27,28,29,30,32,33,34,35,36,56,63,124]. As noted in [125], data-driven fault detection using sensor-based monitoring is crucial for ensuring the safe and reliable operation of railway point machines, despite practical deployment challenges.

Substantial progress has been made in understanding the physics of cable degradation and developing diagnostic techniques to assess cable health. A wide range of sensing technologies has been proposed for detecting PD activity, including capacitive couplers, magneto-resistive sensors [1], piezoelectric acoustic detectors [11], fiber-optic sensors [18], and radio-frequency probes [6,21]. Advanced signal processing techniques, such as wavelet denoising [13] and time–frequency domain reflectometry [5], have improved the accuracy of PD detection and localization under noisy field conditions.

Meanwhile, machine learning (ML) and deep learning (DL) techniques have been applied to classify fault types, predict remaining useful life, and reconstruct PD signals [7,9,14,16,28]. Numerical simulations using finite element methods (FEM) have further enhanced our understanding of electric field distributions, space charge dynamics, thermal effects, and stress cone behavior in joints and terminations [75,84,98].

Despite these advances, several gaps remain. Existing studies often focus on specific technical aspects—such as a particular PD detection method or a single type of aging model—without offering a comprehensive view that integrates sensing, signal interpretation, physical modeling, and maintenance planning. Moreover, there is a lack of cohesive analysis that bridges laboratory-scale investigations with large-scale field deployments. The unique challenges of HVDC cable systems—such as polarity reversals, space charge accumulation, and long-duration transients—are only partially addressed in the current literature and demand more unified treatment. Additionally, practical issues like measurement uncertainty, calibration standards, and cost-effective sensor deployment strategies are underrepresented in many academic discussions.

Given the increasing reliance on underground and submarine cable networks—particularly in high-renewable power systems, offshore wind farms, and interregional HVDC interconnectors—ensuring the long-term reliability and safety of these assets is a critical engineering priority. At the same time, advances in sensing, computing, and data analytics now allow for unprecedented monitoring and diagnostic capabilities. This creates a compelling opportunity to synthesize disparate threads of research into a unified, actionable framework for cable reliability assessment and failure mitigation.

The main objectives of this review are therefore fourfold:

- To provide a systematic and multidisciplinary overview of the most relevant and recent developments in condition monitoring, fault detection, lifetime estimation, and insulation modeling of HV/MV cable systems.
- To identify and analyze the strengths, limitations, and applicability of various sensor technologies, signal processing methods, machine learning algorithms, and physical aging models under real-world operating conditions.
- To explore the challenges and opportunities associated with HVDC cable systems, including space charge effects, electric field distortion, polarity reversals, and insulation degradation under non-uniform thermal stress.
- To bridge the gap between theory and practice by highlighting implementation issues such as measurement uncertainty, sensor deployment strategies, calibration methodologies, and the role of emerging standards (e.g., IEC 60270).

Through this integrated approach, the review aims to serve as both a technical reference and a strategic guide for researchers, engineers, utilities, and manufacturers involved in the design, operation, and maintenance of power cable infrastructure.

This article presents a comprehensive and critical review of the state-of-the-art techniques and models used in the diagnosis, modeling, and lifetime management of power cable systems. The scope encompasses:

- Condition monitoring (CM) methods for high-voltage cables, focusing on PD detection and sensor technology.
- Aging mechanisms in XLPE insulation, including thermal, electrical, mechanical, and environmental stressors, under both AC and DC conditions.
- Signal processing and machine learning frameworks for fault detection, classification, and location, including hybrid physics-informed models.
- Numerical modeling and simulation techniques for PD behavior, space charge accumulation, and electric field distortion.
- HVDC-specific challenges such as polarity reversal, space charge behavior, and thermal gradient-induced degradation.
- Testing and measurement protocols, including uncertainty analysis, sensor calibration, and international standardization efforts.
- Maintenance planning strategies, including condition-based maintenance (CBM), predictive maintenance (PdM), and reliability-centered scheduling under uncertainty.

**Figure 1:**
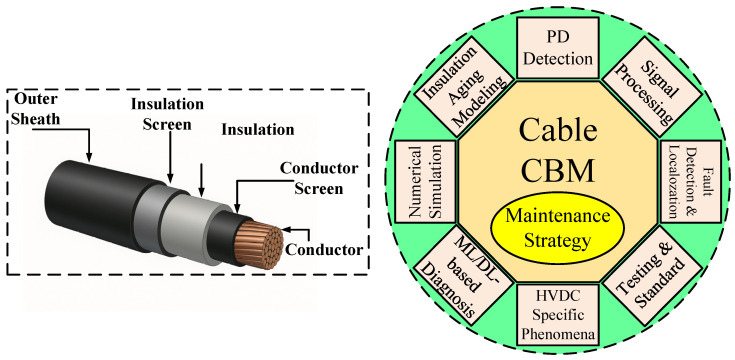
Schematic overview of high-voltage XLPE cable structure and associated analytical modules.

A conceptual diagram is provided in Figure 1 to illustrate the general structure of an XLPE-insulated high-voltage cable along with the key diagnostic and analytical domains addressed throughout this review. The figure serves as a roadmap connecting physical degradation phenomena, modeling techniques, sensing technologies, and maintenance frameworks.

By combining insights from these domains, the review aims to provide a holistic framework for cable asset management that supports the transition toward smarter, more resilient, and more predictive power systems.

A structured literature-screening methodology was adopted to ensure the transparency and representativeness of this review. The search covered publications using focused keywords related to PD detection, XLPE aging, HVDC insulation behavior, FEM-based modeling, and ML/DL diagnostics. Studies were selected based on predefined inclusion/exclusion criteria, duplicates were removed, and a PRISMA-style workflow guided the screening process to guarantee comprehensive coverage of both foundational and recent advancements. This article not only summarizes existing approaches but also provides a critical evaluation of their strengths, limitations, and practical applicability, thereby extending the scope from a descriptive overview to a comprehensive technical review.

To contextualize the contribution of this work, Table 1 contrasts (i) the motivation for conducting this review and (ii) the key differentiating elements compared with existing surveys on HV/MV cable diagnostics. This structure clarifies the unique integration achieved here between HVDC-specific insulation phenomena and advanced diagnostic/monitoring methodologies.

The remainder of the article is organized as follows:

➢ Section 2 reviews condition monitoring strategies, with emphasis on PD detection technologies, sensor types, and signal acquisition techniques.
➢ Section 3 investigates insulation aging mechanisms and lifetime estimation methods for XLPE-insulated cables under various electrical and thermal stressors.
➢ Section 4 presents advanced fault detection and localization techniques, comparing time-domain, frequency-domain, and hybrid diagnostic approaches.
➢ Section 5 explores the modeling and simulation of PD phenomena, integrating both physical and data-driven models.
➢ Section 6 analyzes insulation behavior in AC and DC environments, including environmental degradation, space charge effects, and transient voltage stresses.
➢ Section 7 provides an in-depth examination of numerical simulation tools, including FEM-based electric field and thermal modeling of cable components.
➢ Section 8 discusses the application of machine learning and deep learning for PD classification, fault diagnosis, and predictive maintenance.
➢ Section 9 focuses on HVDC cable systems, reviewing life estimation models, geometric optimization, and advanced insulation designs under polarity reversal.
➢ Section 10 summarizes testing protocols and standards, with particular attention to measurement accuracy, calibration, and emerging methodologies.
➢ Section 11 reviews intelligent maintenance strategies, including CBM, PdM, asset life modeling, and optimization under uncertainty.
➢ Finally, Section 12 presents the conclusions and outlines potential future directions for research and development in this critical area.

## 2. Condition Monitoring

The integrity and operational longevity of HV and MV cable systems are central to the resilience of modern power networks. As these assets are exposed to prolonged electrical stress, environmental factors, and thermal cycles, their insulation systems become susceptible to degradation and eventual failure. Traditional time-based maintenance approaches often fall short in capturing the complex and stochastic nature of such failures. In contrast, CM offers a more intelligent and proactive approach by enabling continuous or periodic observation of cable health in situ. Through systematic collection and interpretation of electrical, acoustic, thermal, and electromagnetic signatures, CM not only facilitates early detection of developing faults but also provides the data foundation for predictive maintenance, reliability assessment, and lifetime modeling.

Recent developments in sensing technologies, signal processing algorithms, and artificial intelligence have significantly advanced the capabilities of CM systems. These systems now encompass a broad range of diagnostic modalities and are increasingly embedded into digital substations, smart grids, and HVDC links. This section presents a detailed and structured overview of state-of-the-art CM methodologies, covering PD-based techniques, sensor technologies, signal modeling, environmental challenges, machine learning integrations, and scalable monitoring platforms.

### 2.1. Objectives the Rationale and Evolution of Condition Monitoring

Condition monitoring in power cable systems is no longer a luxury but a necessity. As highlighted in [19], modern power utilities face growing demand for grid availability and system reliability, particularly with aging infrastructure and increasing renewable penetration. CM enables utilities to transition from reactive to proactive asset management by identifying anomalies in insulation performance long before failure occurs.

According to the editorial overview in [30], effective CM systems must fulfill four fundamental criteria: they should be non-invasive, continuous, accurate, and predictive. These criteria reflect both the practical limitations of on-site diagnostics and the high expectations of modern asset managers. The importance of integrating CM within a broader digital ecosystem—where field measurements feed into centralized analytics platforms—is also emphasized in [29], reinforcing the idea that CM is foundational to digital asset management.

### 2.2. PD as a Cornerstone of CM

Among various diagnostic phenomena, PD remains the most informative and direct indicator of insulation health. PD refers to a localized electrical breakdown that does not completely bridge the electrodes. Its occurrence, intensity, and pattern can provide early insights into the progression of insulation defects such as voids, delamination, and water trees.

#### Sensor Technologies for PD Detection

**Figure 2:**
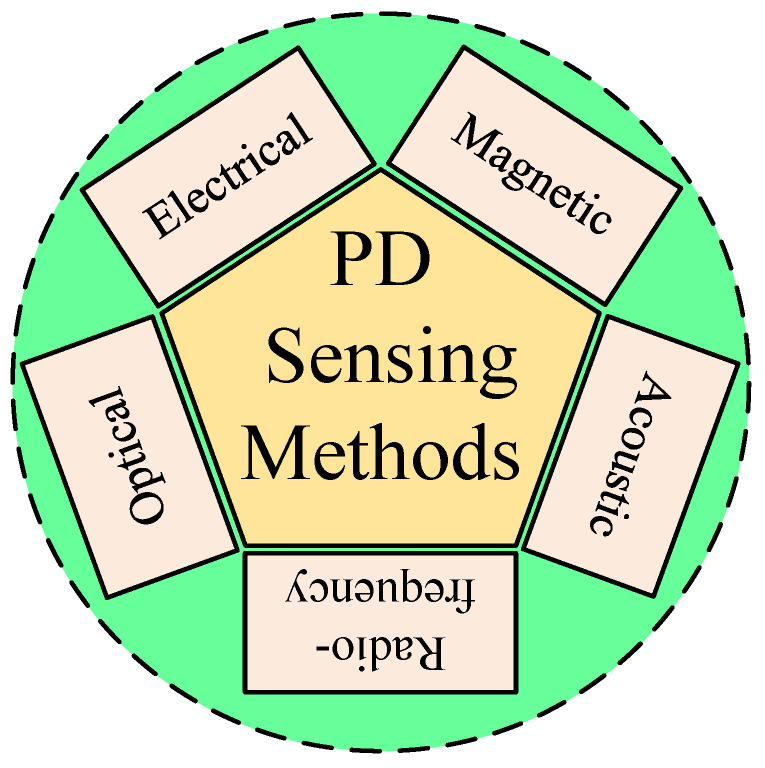
PD sensing methods classifications.

Over the past decade, numerous PD sensing methods have been developed and optimized. Figure 2 provides a taxonomic overview of these sensing technologies, categorized by physical domain, signal type, and application context.

Figure 2 classifies PD sensing methods into five categories:

- Electrical (capacitive couplers, current transformers).
- Magnetic (magneto-resistive sensors [1]).
- Acoustic (piezoelectric sensors [11]).
- Optical (fiber Bragg gratings and fluorescent fiber [18,24]).
- Radio-frequency (inductive loops and antennas [6,21]).

Each sensing modality presents trade-offs between sensitivity, spatial resolution, cost, and environmental immunity. For instance, magneto-resistive sensors [1] offer high sensitivity to magnetic field transients but require shielding to mitigate electromagnetic interference. Optical fiber sensors [24] provide distributed sensing capability over long distances, particularly advantageous in HVDC and submarine cable applications, but they are costlier and complex to deploy.

Acoustic sensors, such as those investigated in [11], are particularly useful in joints and terminations where PD may not generate strong electrical transients. However, the accuracy of such measurements is highly dependent on sensor placement and mechanical coupling with the cable surface.

### 2.3. Signal Processing, Modeling, and Localization Techniques

While PD sensors provide the raw data, the real diagnostic power lies in how these signals are processed and interpreted. Modern CM systems employ a suite of advanced signal processing algorithms to extract useful features from noisy data.

Wavelet transform-based denoising has emerged as a robust technique to isolate PD pulses in environments rich with harmonic and switching noise. In [13], a thorough analysis of mother wavelet selection is presented, revealing how the effectiveness of denoising depends on the spectral content of both PD and background noise. Adaptive selection of wavelet functions is proposed to improve detection reliability under variable field conditions.

In terms of fault localization, techniques such as Time–Frequency Domain Reflectometry (TFDR) and Electromagnetic Time Reversal (EMTR) have gained traction. As described in [5], TFDR involves sending a broadband signal through the cable and analyzing reflected signals to detect impedance discontinuities. The reflected voltage signal is modeled as:

(1) $$V_{r}\left(t\right)=V_{i}\left(t\right)+\sum_{k=1}^{n}\Gamma_{k}V_{i}\left(t-2\tau_{k}\right)$$

Here, Γ*_k_* is the reflection coefficient at point k, and τ_k_ is the round-trip time delay. This allows estimation of the fault location using known signal propagation velocities.

The EMTR technique [3] uses the time-reversed version of the recorded PD signal to backtrack its source location. This method is particularly effective in complex networks with multiple reflection points, as it focuses energy back to the emission site, even in the presence of interfering reflections.

### 2.4. Advanced Fault Location Under Complex Conditions

In realistic scenarios, cables may exhibit multiple insulation defects simultaneously. Conventional PD detection algorithms often fail under such conditions due to overlapping waveforms and spatial aliasing. In [4], a novel joint analysis of the reflection coefficient spectrum and matched filter matrices was proposed to detect and localize multiple faults. This method achieves superior accuracy by combining spectral and temporal signal domains.

Further, ref. [17] introduced an improved MUSIC (Multiple Signal Classification) algorithm tailored for transformer PD localization, achieving high spatial resolution even with low SNR measurements. The robustness of these methods under noise, sensor misalignment, and parameter drift highlights the necessity of intelligent localization schemes in modern CM.

**Figure 3:**
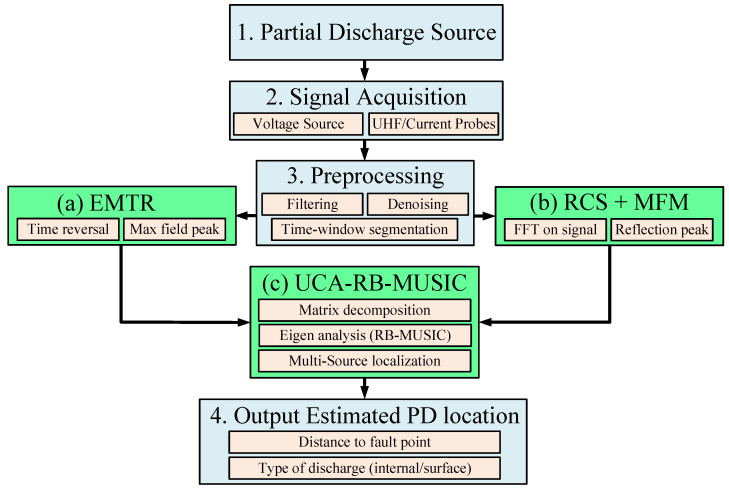
PD detection and localization techniques.

Figure 3 presents a comparative analysis of localization error across TFDR, EMTR, and MUSIC-based methods under various SNR levels and cable configurations. It demonstrates that joint signal-domain methods outperform single-domain approaches, particularly when fault spacing is minimal.

### 2.5. Environmental and Operational Influences on Measurement Accuracy

One of the key challenges in real-world CM systems is the influence of external environmental variables. In [25], it was shown that increasing ambient temperature leads to intensified PD activity, likely due to changes in dielectric permittivity and thermal stress. If uncorrected, such environmental effects can lead to false diagnostics or unnecessary maintenance actions.

Similarly, ref. [12] modeled the uncertainty in PD measurements using statistical inference and Monte Carlo simulations, accounting for environmental interference and equipment tolerance. The results suggest that uncertainty levels must be included in any quantitative interpretation of CM data.

The sensitivity of acoustic-based PD measurements to sensor placement was validated in [11], where even slight deviations from the optimal position significantly altered measured waveforms, leading to errors in time-of-flight localization. These insights reinforce the need for adaptive calibration and context-aware interpretation in field applications.

### 2.6. Intelligent Diagnostics via Machine Learning and Deep Learning

Traditional CM systems rely on expert-driven thresholding and rule-based interpretation. However, the increasing volume and complexity of sensor data have necessitated a shift toward data-driven diagnosis, where machine learning (ML) and deep learning (DL) play transformative roles.

In [7], a fused model combining Markov Transition Fields (MTF) and Gramian Angular Fields (GAF) with multihead attention mechanisms was developed, offering a 96.2% fault classification accuracy. Similarly, ref. [9] proposed a hybrid BiGRU–ResNet–MA model to identify faulted phases in three-core cables, achieving over 97% accuracy using ground wire current features.

Multiple studies have demonstrated the effectiveness of DL in PD classification:

- CNN-based classifiers for raw signal images [8];
- Attention-enhanced ultrasound PD identification [14];
- Hybrid LSTM-GRU structures for GIS and overhead line PD patterns [15,16].

Table 2 compares several DL-based fault detection models from recent literature, summarizing architecture type, feature extraction methods, and performance metrics. This table illustrates the superior scalability and generalization capability of hybrid temporal–spatial deep learning models compared to traditional SVM or threshold-based techniques.

### 2.7. Scalable, Cost-Efficient CM Systems and Predictive Integration

Despite the technological advancements, large-scale deployment of CM systems remains constrained by economic and logistical factors. In [20], a compact, low-cost PD monitoring unit for MV joints was introduced, demonstrating acceptable diagnostic accuracy while significantly reducing system cost.

More importantly, CM systems are increasingly being integrated into maintenance planning algorithms. Studies such as [22,33,35] show how CM-derived health indicators can be used to prioritize maintenance activities, defer unnecessary interventions, and manage budget allocations. Also, ref. [34] proposed an Overall Condition Index (OCI) for pipeline diagnostics, which—when powered by neural networks—can be extended to cable asset management as well.

## 3. Lifetime Estimation and Aging Analysis

The service life of HV and MV power cables—especially those insulated with XLPE—is predominantly determined by complex interactions among electrical, thermal, mechanical, and environmental stressors. Over time, these factors lead to insulation deterioration through mechanisms such as thermal oxidation, space charge accumulation, PD, electrical treeing, and water treeing. Accurate lifetime estimation is therefore essential for ensuring operational reliability, preventing catastrophic failures, and enabling cost-effective maintenance and replacement strategies.

This section presents a comprehensive overview of aging mechanisms, accelerated aging models, mathematical formulations, and experimental insights into insulation lifetime estimation, with a focus on XLPE cables under both AC and DC stress conditions. Additionally, effects of installation environment, load cycling, and material additives are addressed in depth.

### 3.1. Thermal Aging and Oxidative Degradation of XLPE

Thermal aging is one of the most dominant degradation mechanisms in XLPE cables, especially under long-term elevated temperatures. As shown in [38], thermal exposure leads to molecular chain scission and crosslink bond degradation, catalyzed by oxygen diffusion. The degradation kinetics can be described using the Arrhenius model:

(2) $$k\left(T\right)=A\cdot e^{-\frac{E_{a}}{RT}}$$

where k(T) is the rate of degradation, A is the pre-exponential factor, E_a_ is the activation energy (eV), R is the gas constant, T is the absolute temperature (K). The lifetime L at a given temperature can thus be predicted as:

(3) $$L\left(T\right)=\frac{1}{k\left(T\right)}=\frac{1}{A\cdot e^{-\frac{E_{a}}{RT}}}$$

In [45], a novel genetic algorithm optimization of Arrhenius coefficients was proposed to enhance the lifetime prediction accuracy, using insulation resistance and breakdown strength as fitness functions. The study demonstrated improved model convergence over traditional arithmetic regression approaches.

**Figure 4:**
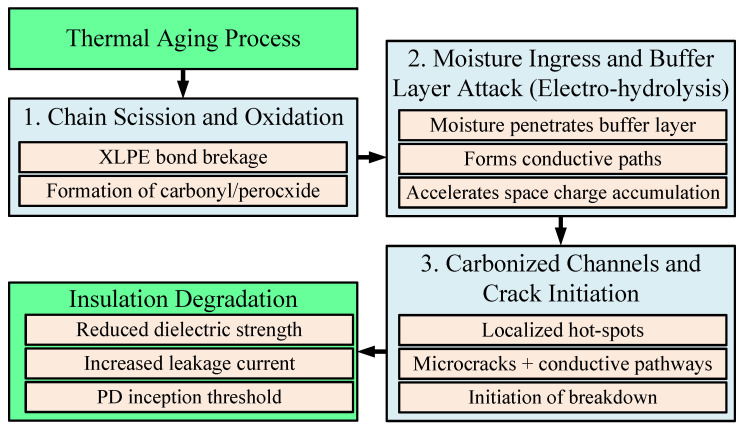
Mechanisms of thermal aging and insulation degradation in XLPE cables.

Figure 4 shows insulation resistance decline over aging time for thermally aged XLPE samples under different antioxidant treatments, revealing the beneficial effects of stabilizers in slowing down oxidative aging.

### 3.2. Electrical Aging and Field-Induced Degradation

Electrical aging arises from continuous or pulsed electric field exposure, leading to charge injection, space charge formation, and eventually electrical tree initiation. According to [37], the electrical lifetime under uniform electric stress EEE follows an inverse power law relationship:

(4) $$L\left(E\right)=L_{0}{\left(\frac{E_{0}}{E}\right)}^{n}$$

where L_0_ is the reference lifetime under E_0_, n is the field acceleration factor, typically ranging between 5 and 20 for XLPE.

Experimental data from [37,43] showed that XLPE lifetime under AC stress is significantly lower when PD is active, particularly for insulation near accessories and terminations. Also, ref. [43] further revealed that fluoropolymer insulations show higher PD inception thresholds and slower aging under identical test conditions, indicating a potential for improved lifetime in harsh environments.

Temperatures are expressed in kelvin (K), electric field in MV m^−1^, activation energy in kJ mol^−1^, and lifetime in hours unless stated otherwise. Table 3 summarizes typical lifetime-reduction trends under different operational stressors and material conditions, based on consolidated findings from [37,38,39,40,41,42,43,44,45,46,47,48,49,50,51,52,53,54,55,56].

**Figure 5:**
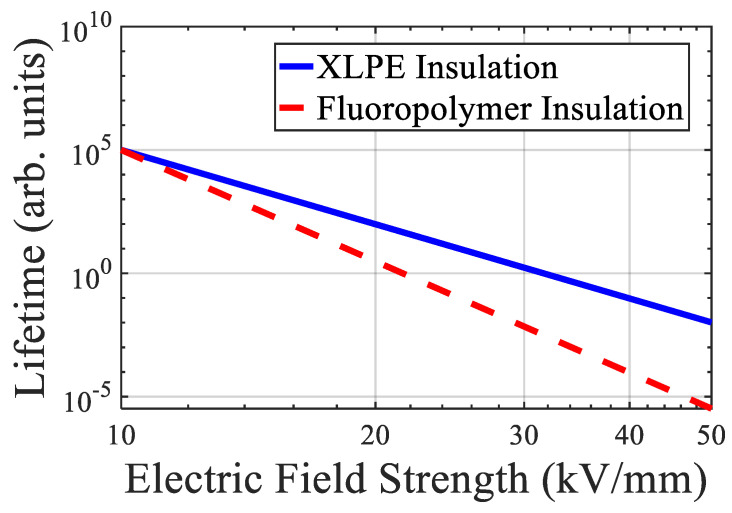
Lifetime vs. electric field strength.

Figure 5 compares lifetime versus electric field strength for XLPE and fluoropolymer-based insulation, showing distinct slopes and breakdown profiles.

### 3.3. Lifetime Under DC Stress and Space Charge Effects

In HVDC applications, electrical aging is aggravated by space charge accumulation due to unipolar injection and weak recombination. Studies such as [39,40,48] demonstrate that space charge profiles significantly distort the electric field distribution over time, leading to localized over-stressing and premature breakdown.

To model this, refs. [49,54] proposed a field-temperature-coupled model of DC conductivity σ(E,T), given by:

(5) $$\sigma \left(E,T\right)=\sigma_{0}\cdot e^{\alpha E}\cdot e^{-\frac{E_{a}}{RT}}$$

where α is the electric field coefficient of conductivity. By integrating this into Maxwell’s equations and Poisson’s equation, one can simulate electric field reversal under polarity change events [39].

Ref. [40] applied the DMM (Double Multi-stress Model) for life estimation under cyclic DC load conditions. The model calculates time-to-failure using a damage accumulation approach based on a load-history matrix.

### 3.4. Annealing, Recovery, and Material Rejuvenation

Interestingly, XLPE insulation exhibits partial recovery under thermal annealing. According to [46], retired HV cables subjected to post-operation annealing showed improvements in insulation resistance and dielectric loss factor, attributed to recrystallization and diffusion of residual by-products.

A similar observation in [44] led to the development of a resistance recovery function:

(6) $$R\left(t\right)=R_{0}\cdot \left(1-e^{\lambda t}\right)$$

where λ is the annealing recovery rate constant. This behavior is particularly relevant for retrofitting aged cables with thermal stabilization treatments.

### 3.5. Environmental and Installation-Dependent Aging

Installation conditions—such as soil temperature, humidity, proximity to thermal sources—can accelerate cable aging. In [41], a study on service-aged cables installed near CHP (Combined Heat and Power) plants revealed localized degradation zones with decreased elongation-at-break (EAB) and increased oxidative indices.

Furthermore, ref. [53] demonstrated that seasonal thermal cycling induces mechanical stress at insulation–semiconductor interfaces, promoting micro-voiding and early water tree formation. The estimated reduction in service life under heavy cycling was up to 25%.

Table 4 summarizes the key findings from aging studies under various operational conditions and materials.

### 3.6. Advanced Modeling for Life Prediction and Design

Recent contributions [49,51,52] emphasize the need for multi-physics modeling combining electrical, thermal, and mechanical domains. For example, ref. [51] introduced a life-based geometric optimization model to determine optimal insulation thickness, semi-conductive layer configuration, and conductor size. The optimization goal is maximizing lifetime L subject to stress, field, and loss constraints.

Reference [52] extended this to transient conditions, including fault-induced heating and switching surges. Using finite element simulations, it was shown that optimized cable designs could increase service life by 40% without increasing total mass or cost.

## 4. Fault Detection and Localization

The reliability of power systems critically depends on the early and accurate detection and localization of cable faults. Whether the cable is installed underground, underwater, or in industrial installations, faults such as insulation breakdown, conductor short, reverse grounding, and PD can lead to prolonged outages, safety risks, and costly repairs. This section presents an in-depth review of traditional and advanced methods for fault identification and localization, including signal-based, machine learning, and hybrid model approaches.

### 4.1. Overview of Cable Fault Types and Signatures

Power cable faults can be broadly categorized based on the nature of the defect and its temporal behavior:

- Permanent Faults: e.g., conductor breakage, insulation breakdown.
- Intermittent Faults: e.g., incipient thermal defects, water trees.
- PD-Induced Faults: progressive dielectric degradation.
- High-Impedance Faults: weak insulation or corroded joints.

Each fault type presents unique electrical signatures such as reflection coefficients, traveling wave anomalies, leakage currents, or PD pulses.

**Figure 6:**
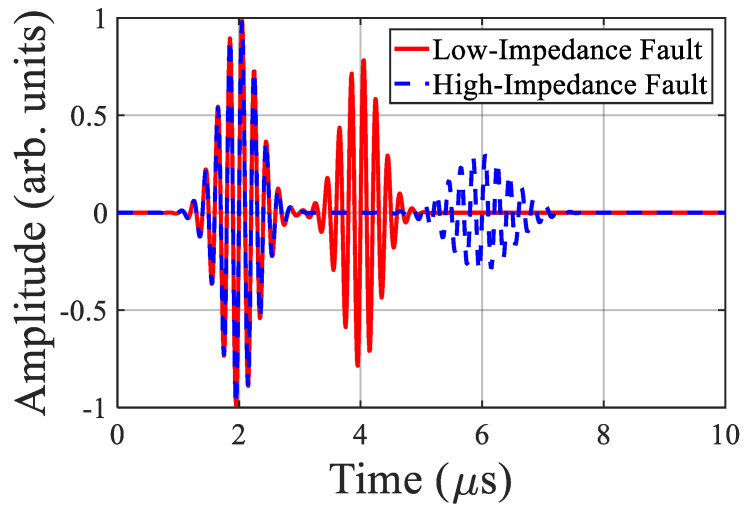
TDR signals for low- and high-impedance faults.

Figure 6 illustrates typical time-domain reflectometry (TDR) signals for low- and high-impedance faults, showing delayed reflections and waveform attenuation characteristics.

### 4.2. Reflectometry-Based Methods

TDR and FDR are widely used for fault location in shielded and unshielded cables. According to [5], the TDR reflection coefficient Γ for a fault at position x can be calculated as:

(7) $$\Gamma \left(x\right)=\frac{Z_{f}\left(x\right)-Z_{0}}{Z_{f}\left(x\right)+Z_{0}}$$

where Z_0_ is the characteristic impedance of the cable, Z_f_(x) is the impedance at the fault point.

The fault distance d can then be determined using the round-trip time t and signal propagation velocity v:

(8) $$d=\frac{v\cdot t}{2}$$

In [4], a joint analysis of the reflection coefficient spectrum and a matched filter matrix was proposed to enhance localization precision for multiple faults. The matched filter output y[n] for an incoming signal x[n] is given by:

(9) $$y\left[n\right]=\sum_{k=0}^{N-1}x\left[k\right]\cdot h\left[n-k\right]$$

where h[n] is the matched template corresponding to known fault signatures. This method allows for resolving closely spaced faults with overlapping reflections.

**Figure 7:**
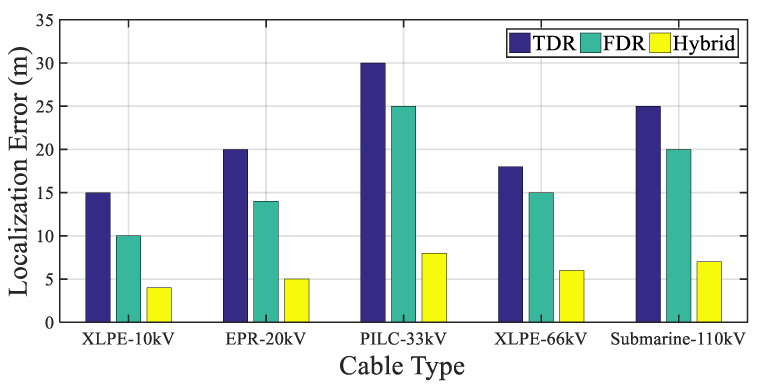
Fault localization accuracy comparison.

Figure 7 compares fault localization accuracy of conventional TDR, FDR, and the proposed hybrid method across multiple cable types.

To clarify the basis of the localization error values reported in Figure 7, the results correspond to a unified simulation scenario applied to all cable types. A low-impedance insulation fault was placed 1.2 km from the sending end on a 2 km line, and the electrical parameters (propagation velocity and attenuation) were assigned according to the dielectric properties of each cable type (XLPE–10 kV, EPR–20 kV, PILC–33 kV, XLPE–66 kV, and Submarine–110 kV). The TDR method employed a 1–50 MHz broadband excitation pulse, while FDR used a 10 kHz–5 MHz frequency sweep; the Hybrid method combines the broadband TDR excitation with frequency-domain correlation. Localization error was computed by comparing the estimated distance from the reflectogram peak (TDR), impedance–frequency signature (FDR), or hybrid correlation output with the known fault location. Representative reflectograms were used during the evaluation, even though they are not shown in the figure, to ensure consistent and traceable extraction of the localization error values.

### 4.3. PD Detection and Localization

PD is a precursor to insulation failure, and its localization is essential for proactive maintenance. In XLPE cables, PD signals exhibit high-frequency transients, often masked by noise.

Reference [59] introduces an improved Generalized Cross-Correlation (GCC) algorithm for PD localization using multiple sensors. Given two signal streams s_1_(t) and s_2_(t), the GCC function is:

(10) $$R_{12}\left(\tau \right)=\int S_{1}^{*}\left(f\right)\cdot S_{2}^{*}\left(f\right)\cdot \psi \left(f\right)e^{j2\pi f\tau }df$$

where S_1_(f) and S_2_(f) are the Fourier transforms of the input signals, and Ψ(f) is a weighting function that enhances PD correlation over noise.

Table 5 compares time resolution and localization error of various PD-based techniques including UHF, acoustic, and GCC methods [24,59,60]. To ensure accuracy and transparency, the localization error values reported in Table 5 were checked against the original studies. In cases where the numerical error was not explicitly provided, the entry is marked as “Not explicitly reported.” It is also important to note that differences in measurement bandwidth, sensor type, and time resolution among these studies significantly influence localization performance; therefore, direct comparison across references should be interpreted with caution.

### 4.4. Deep Learning-Based Fault Diagnosis

With the rise in high-resolution measurement and sensor data, deep learning (DL) methods have shown great promise in cable fault diagnosis and classification.

In [8], a deep convolutional neural network (DCNN) trained on simulated and real-world fault data achieved 98% accuracy in multi-class fault classification. The input was a time–frequency spectrogram derived using continuous wavelet transform (CWT):

(11) $$W\left(a,b\right)=\int x\left(t\right)\cdot \psi^{*}\left(\frac{t-b}{a}\right)dt$$

where ψ is the mother wavelet, and a, b denote scale and translation.

Similarly, ref. [9] developed a BiGRU-ResNet-MA hybrid model for fault line identification in three-core cables, leveraging ground wire current patterns. This model integrates the bidirectional gated recurrent unit (BiGRU) for temporal dependency and ResNet blocks for feature enhancement.

### 4.5. Optical and Acoustic Sensing Techniques

Optical and ultrasonic sensors have emerged as non-invasive fault detection solutions, particularly in long-distance XLPE installations.

In [24], an optical fiber sensor system was employed to localize PD sources based on ultrasonic signal delay:

(12) $$d=\frac{v_{ultra}\cdot \Delta t}{2}$$

where Δt is the differential time-of-arrival and v_ultra_ is the ultrasonic propagation velocity in the cable medium.

Reference [62] further introduced adversarial denoising autoencoders to reconstruct clean PD signals from noisy ultrasonic recordings, significantly improving detection rate in noisy substations.

### 4.6. Hybrid and Intelligent Methods

Recent efforts have focused on integrating multiple domains (signal, frequency, thermal, spatial) into hybrid fault diagnosis frameworks:

- Ref. [58] proposed a probabilistic CNN combined with discrete wavelet transform (DWT) for high-fidelity feature extraction.
- Ref. [63] used feedforward neural networks for fault type and location prediction using voltage and current waveform snapshots.
- Ref. [64] presented a SVM-TT transform-based method for locating faults in hybrid overhead-underground transmission systems.

Table 6 summarizes the key features, input data, and performance metrics of these AI-powered diagnostic systems.

For consistency and transparency, the accuracy values in Table 6 were verified based on the original references. When a study explicitly reported quantitative accuracy (e.g., classification accuracy, detection success rate, or validation performance), that value is directly included. In cases where accuracy was not numerically stated or only qualitative performance indicators were provided, the entry is marked as “Not explicitly reported.” It should also be noted that accuracy metrics across different studies are based on varying datasets, sensor types, and evaluation protocols, and therefore cannot be directly compared without considering these methodological differences.

### 4.7. Challenges and Future Directions

Despite substantial progress in monitoring, diagnostics, aging analysis, and predictive maintenance of HV/MV XLPE cable systems, several unresolved challenges remain that limit large-scale and reliable field deployment. First, PD localization under multi-reflection conditions continues to suffer from noise sensitivity, dispersion effects, and inconsistent sensor bandwidths. Next-generation diagnostic systems will require hybrid multi-physics models combined with adaptive ML techniques to achieve sub-meter accuracy in complex networks.

Second, space-charge characterization in HVDC insulation remains fundamentally constrained by incomplete understanding of charge injection dynamics, trap distributions, and polarity-reversal transients. Existing models often lack validation against full-scale cable systems. Future research should focus on standardized HVDC stress protocols, unified material models, and in situ charge measurement technologies.

Third, ML/DL-based diagnostic models face issues of dataset scarcity, domain shift between laboratory and field conditions, and limited interpretability. Emerging directions include physics-informed neural networks, self-supervised learning, federated training across utilities, and explainable AI frameworks to ensure traceability of diagnostic decisions.

Fourth, aging models remain insufficiently coupled, typically isolating thermal, electrical, mechanical, and environmental stressors. Multi-stress accelerated aging platforms, validated by real service data, are needed to establish reliable lifetime prediction and maintenance optimization methods.

Fifth, integration of cable diagnostics into digital asset management ecosystems remains fragmented. Future developments should enable seamless fusion of PD, thermal, mechanical, and operational data into digital twins capable of near-real-time health forecasting and maintenance orchestration.

Finally, the field requires stronger standardization efforts to harmonize measurement bandwidths, uncertainty quantification practices, sensor calibration procedures, and evaluation metrics. Such harmonization will enable reproducible benchmarking and accelerate industrial adoption.

Overall, these challenges outline a roadmap for future research that links advanced diagnostics, computational modeling, intelligent algorithms, and asset management strategies into a cohesive, scalable, and field-ready framework—representing a key contribution this review seeks to consolidate.

## 5. PD and Its Modeling

PD is one of the most critical indicators of insulation degradation in high-voltage cable systems. It represents a localized dielectric breakdown of a small portion of the insulation under high electric stress, which does not bridge the electrodes completely. PD activity leads to gradual erosion of insulation material and eventually causes complete dielectric failure. This section thoroughly analyzes the origins, detection, modeling, and simulation of PD phenomena in XLPE-insulated cables, integrating both physical models and data-driven techniques.

### 5.1. Fundamentals of PD in Cable Insulation

PDs occur due to inhomogeneities in the insulation system, such as voids, cracks, impurities, or sharp protrusions, where the local electric field exceeds the breakdown strength of the dielectric. In XLPE-insulated cables, voids between insulation layers or around semi-conductive shields are typical PD sources [1,43].

The inception voltage of PD is given by:

(13) $$V_{PD}=\frac{d}{\varepsilon_{r}}\cdot \sqrt{2\varepsilon_{0}\gamma E_{BD}}$$

In this expression, d represents the effective thickness across the dielectric gap, ϵ_r_ is the relative permittivity of the insulation material, ϵ_0_ is the vacuum permittivity, γ is the surface energy associated with the dielectric interface, and E_BD_ denotes the breakdown electric field strength specific to the material. The formula provides insight into how intrinsic material properties and defect geometry influence the threshold voltage at which PD begins.

**Figure 8:**
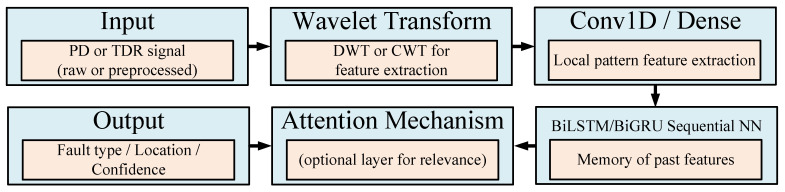
Block diagram of deep neural network architecture.

Figure 8 illustrates the electric field distribution within an XLPE cable segment containing a void. As shown, the void significantly distorts the uniformity of the field, concentrating stress at the void’s edges.

### 5.2. Detection Methods for PD

Several techniques have been developed to detect PD activity, each leveraging different physical domains:

- Electrical Detection: This traditional method involves direct measurement of transient current pulses generated by PD events. While it offers high sensitivity, it is also prone to noise and electromagnetic interference [1,60].
- Ultrasonic/Acoustic Emission (AE): PD activity generates mechanical stress waves that can be detected acoustically. This method is particularly useful for GIS equipment and XLPE joints, although its detection range is relatively limited [11,14].
- Electromagnetic Detection (UHF/VHF): This approach uses antennas to capture high-frequency electromagnetic emissions from PD sources. It is highly sensitive and suitable for compact installations like gas-insulated substations (GIS) and cable terminations [6,80].
- Optical Sensing: Recent advancements include the use of fiber-optic sensors and two-dimensional material-based sensors to detect PD in confined or early-stage conditions [18].

Table 7 offers a comparative overview of these detection methods, highlighting their relative sensitivity, noise immunity, detection range, and suitable applications.

### 5.3. Modeling of PD: Physical and Data-Driven Approaches

The behavior of PD is highly nonlinear and requires advanced modeling for reliable interpretation and simulation. Two primary modeling strategies exist: physical (deterministic) models and data-driven (statistical or machine learning) models.

#### 5.3.1. Physical Models of PD (Void-Based Modeling)

A widely accepted physical model is the so-called ABC model, which considers the discharge to occur inside a void of known geometry embedded within a solid dielectric. Under this model, the apparent charge q_a_ generated during each PD event is given by:

(14) $$q_{a}=C_{eq}\cdot \left(V_{m}-V_{d}\right)$$

Here, C_eq_ is the equivalent capacitance of the discharge path, V_m_ represents the peak applied voltage across the insulation, and V_d_ is the voltage at which the breakdown occurs across the void. This model enables the simulation of electrical field stress and discharge energy, particularly when integrated with finite element analysis tools.

The simulation highlights how the electrical field intensifies around the void edges, resulting in localized current concentration. This effect is a critical precursor to insulation degradation, consistent with observations reported in [75].

#### 5.3.2. Data-Driven and Hybrid Models

In parallel with physical modeling, data-driven approaches based on machine learning have emerged as powerful tools for PD pattern recognition, classification, and localization. Recent studies have demonstrated the effectiveness of the following models:

- Convolutional Neural Networks (CNN): For spatial pattern recognition in PD signals [70].
- Bidirectional LSTM-GRU Networks: To capture temporal dependencies in sequential PD data [16].
- Generative Adversarial Networks (GANs) and Autoencoders: For signal reconstruction and denoising, especially in noisy environments [62].
- Hybrid Models: These combine physical insights (e.g., signal propagation physics) with ML-based feature extraction and classification mechanisms [19].

### 5.4. PD Evolution, Electrical Treeing, and Aging

PDs can initiate and accelerate the formation of electrical trees, which are filamentary channels that grow through the insulation material. These trees eventually compromise the dielectric strength and cause failure. The length of an electrical tree as a function of time can be described using the empirical power law:

(15) $$l\left(t\right)=l_{0}+\alpha \cdot t^{n}$$

In this equation, l(t) is the tree length at time t, l_0_ is the initial defect size, and α\alphaα and *n* are material-specific constants derived experimentally. This relationship is useful for estimating the progression of degradation under PD stress conditions in HV cable systems [43,76].

Table 8 summarizes common mathematical models used for representing the growth of electrical trees and the evolution of PD activity.

### 5.5. PD Under DC and Transient Voltage Conditions

Under HVDC conditions, the behavior of PDs becomes more complex due to phenomena such as space charge accumulation, dielectric polarization, and polarity reversal. Unlike AC systems, where PDs tend to occur at voltage peaks, in DC systems the presence of accumulated charge may delay or suppress discharge initiation.

In such cases, a modified version of the PD inception voltage under DC stress is given by:

(16) $$V_{PD}^{DC}=V_{th}+\frac{q_{acc}}{C_{void}}$$

Here, Vth is the theoretical threshold voltage for breakdown, q_acc_ is the accumulated space charge within the void, and C_void_ is the capacitance of the void region. Studies [86,88,89] have shown that external transients or superimposed harmonics can significantly affect V_PD_^DC^ and may result in earlier or delayed discharges depending on the waveform characteristics.

### 5.6. Challenges and Future Trends

Several persistent challenges exist in the modeling and detection of PDs, particularly for long-length underground cables:

- Accurate localization of PD sources in the presence of multiple reflections and noise.
- Differentiation between various PD types, such as internal, surface, and corona discharges.
- Development of real-time diagnostic systems with embedded AI for asset monitoring.
- Integration of multi-physics models accounting for thermal, electrical, and mechanical stress [66,87].

Looking ahead, future directions involve high-resolution 3D simulations of cable joints and terminations, use of transformer-based deep learning models for signal analysis, and the establishment of standardized PD signal datasets for benchmarking machine learning models. Such advancements are critical for building reliable digital twins of power cables that can anticipate failures before they occur.

## 6. Insulation Behavior of Cables

The long-term performance and reliability of high-voltage power cables are fundamentally governed by the electrical, thermal, and environmental behavior of their insulation systems. XLPE has emerged as a widely used insulation material in both HVAC and HVDC applications due to its excellent dielectric properties, thermal resistance, and mechanical flexibility. However, over time, XLPE undergoes degradation due to operational stresses such as temperature cycling, moisture ingress, electrical over-stress, and space charge accumulation. This section explores the mechanisms of insulation aging, the physical and chemical modifications in the material, and the modeling of these changes through analytical and experimental techniques.

### 6.1. Thermal Aging and Antioxidant Degradation in XLPE

Thermal aging is one of the most dominant mechanisms contributing to insulation deterioration. It results primarily from oxidative reactions accelerated at elevated temperatures, which gradually break polymer chains and reduce crosslink density.

Experimental studies [38] demonstrate that aging at 120 °C for several hundred hours causes a significant decline in breakdown strength and insulation resistance, confirming the exponential dependence of degradation rate on temperature.

Antioxidants initially present in XLPE mitigate this process by scavenging free radicals generated during thermal stress. However, as aging progresses, antioxidant concentration diminishes, leaving the polymer more susceptible to thermo-oxidative degradation. This transition from a stabilized to an unstable chemical phase marks the onset of critical deterioration.

### 6.2. Environmental Degradation and Dielectric Performance

Environmental factors such as humidity, installation location, and mechanical loading exacerbate the aging of XLPE insulation. Field data from CHP plant cables [41] show that cables located near heat sources suffered from uneven thermal exposure, resulting in localized embrittlement and oxidation. The dielectric response of such aged insulation is often characterized using the loss tangent:

(17) $$\tan\delta =\frac{\sigma }{\omega \varepsilon }$$

Here, σ represents the electrical conductivity of the insulation, ω = 2πf is the angular frequency of the applied voltage, and ε is the dielectric permittivity. As oxidation and moisture absorption increase conductivity while reducing permittivity, tanδ rises, indicating energy loss and degraded dielectric integrity. Seasonal monitoring in [53] revealed that loss factors peaked during high ambient temperature months, underscoring the combined effect of thermal and environmental aging.

### 6.3. Segmented Aging and Defect Localization

In practical applications, aging does not occur uniformly along the cable but is often segmented due to localized heat generation, mechanical strain, or electrical stress concentration. Segmented thermal aging experiments [42] demonstrate that regions exposed to higher temperatures show more severe dielectric degradation and surface cracking. Using time-domain reflectometry and broadband impedance analysis, these regions can be accurately identified due to abrupt impedance mismatches caused by microvoids, oxidation, and increased conductivity.

The data-driven analysis of defect-prone segments supports predictive maintenance strategies and allows utilities to localize and intervene before catastrophic failure occurs. Moreover, these findings validate the hypothesis that defect localization accuracy strongly correlates with the spatial gradient of the thermal profile.

### 6.4. Space Charge Accumulation and Field Distortion

Under HVDC operation, XLPE insulation exhibits space charge injection from electrodes and subsequent accumulation within the bulk material. This phenomenon leads to significant distortion of the electric field, which is no longer uniform but shaped by local charge densities. The internal electric field E(x) obeys Poisson’s equation:

(18) $$\frac{dE\left(x\right)}{dx}=\frac{\rho \left(x\right)}{\varepsilon }$$

where ρ(x) is the space charge density at position xxx and ε is the local permittivity. Accumulated space charges can locally enhance the electric field beyond the PD inception threshold, triggering internal discharges and initiating electrical treeing.

Studies in [48,95,99] showed that the magnitude and distribution of space charge are affected by insulation temperature, DC polarity reversal, and voltage stress history. In particular, a temperature gradient across the insulation thickness can induce differential mobility of charge carriers, leading to asymmetric space charge profiles and distorted field maps, especially near joints and terminations.

### 6.5. Chemical and Structural Degradation

Chemical degradation in aged XLPE insulation involves oxidation-induced chain scission, reduction in molecular weight, and the formation of polar carbonyl and hydroxyl groups. These chemical alterations, as analyzed through FTIR and DSC in [55], lead to changes in crystallinity and mechanical strength, which in turn affect dielectric breakdown performance. The buffer layer between XLPE and outer sheaths is particularly vulnerable, and its degradation has been linked to localized discharge activity under electro-humid stress [96].

Field-exposed samples also show evidence of microstructural changes such as void formation, interface debonding, and inclusions near protrusions, which act as field enhancers and PD initiation sites [69,91]. As insulation integrity declines, the likelihood of irreversible failure mechanisms like electrical treeing and thermal runaway increases.

### 6.6. Impact of Transients and Polarity Reversal

Seasonal loading and transient over-voltages significantly influence insulation deterioration. Studies such as [53] indicate that seasonal thermal cycling can reduce mechanical integrity by inducing microvoid formation at interface layers, thereby lowering reliability by up to 25%. Transient overvoltage events in HVDC systems—such as switching surges or polarity reversals—trigger PD activity and field localization near stress cones, as reported in [82], increasing the risk of insulation damage during load fluctuations [82]. Table 9 illustrates factors affecting insulation behavior.

### 6.7. Thermal Degradation of Buffer Layers and PET Components

In composite XLPE cable designs, additional layers such as PET buffers contribute to mechanical and electrical insulation. A case study [55] observed that thermo-oxidative degradation of PET buffer layers initiated in conjunction with oxygen presence and elevated temperatures, leading to microcracking, delamination, and eventual PD onset. This degradation underscores the importance of integrated material design and durability assessment in multilayer cable systems.

## 7. Numerical Modeling and Simulation

The study of insulation degradation in power cables—particularly those using XLPE—has significantly advanced through the application of numerical modeling and simulation. These computational approaches provide detailed insights into the evolution of electric fields, temperature profiles, space charge behavior, and PD phenomena, which are otherwise challenging to measure directly. In this section, we synthesize recent developments in modeling tools, simulation frameworks, and validation techniques used to study cable performance under operational and fault conditions.

### 7.1. Electric Field Analysis and PD Simulation in Voids

Localized voids are common initiation sites for PDs and electrical treeing within cable insulation. To capture the behavior of PD pulses and electric field enhancement, finite element modeling (FEM) and time-domain simulations are widely used.

In [75], a 3D axisymmetric FEM model was developed to simulate the electric field and PD pulse formation within a cylindrical void in XLPE insulation under HVDC stress. The simulation captured field enhancement along the void walls, pulse propagation paths, and charge accumulation during each discharge event.

Similarly, ref. [84] modeled electric field behavior in cable segments with multiple gaseous cavities, showing how defect interaction leads to asymmetric field profiles. These simulations provide guidance for insulation design and PD-resistant geometries.

### 7.2. Modeling of Stress Cone Dislocation and Joint Defects

Stress cone dislocations in cable joints are critical failure points, especially in high-voltage systems. In [68], a numerical model of a cable joint with misaligned stress cone geometry was constructed to evaluate PD inception and field distortion. The results showed that even minor angular misalignments led to sharp field gradients and reduced PD inception voltage by up to 30%.

Such modeling supports improved manufacturing tolerances and better diagnostics in cable terminations.

### 7.3. Space Charge and Polarity Reversal Under HVDC Conditions

HVDC cable insulation experiences unipolar charge injection, leading to space charge accumulation and electric field distortion. In [98,99], multi-physics FEM models were constructed to simulate charge transport, polarity reversal, and field distortion under ±500 kV HVDC. These simulations integrated temperature gradients, injection coefficients, and trap densities into Poisson’s and continuity equations:

(19) $$\frac{dE\left(x\right)}{dx}=\frac{\rho \left(x\right)}{\varepsilon }\;and\;\frac{\partial \rho }{\partial t}+\nabla \cdot J=0$$

where ρ(x) is the space charge density and J is the current density, which includes drift and diffusion components.

### 7.4. Thermal Gradient and Water Tree Simulation

Thermal effects significantly influence space charge mobility and defect growth. In [126], a FEM-based model was used to compute electric field and water tree progression in the presence of non-uniform moisture and temperature. The simulation results revealed intensified field lines near wet insulation zones and water trees acting as field enhancers.

Further, ref. [127] employed COMSOL Multi-physics to simulate water tree defects in different cable configurations. The results showed that cables with thicker insulation or improper shielding had higher water tree initiation probabilities [128,129].

### 7.5. Charge Transport and Aging Models in Polymeric Dielectrics

Charge transport modeling is essential for lifetime estimation and dielectric behavior prediction. In [102], a robust numerical method was proposed to simulate bipolar charge injection and recombination using a discretized form of the continuity and Poisson’s equations. This method allows modeling of long-term insulation behavior under both AC and DC stress.

Incorporating this into aging simulation, ref. [37] predicted insulation breakdown life using inverse power law models linked to simulated field stress as Equation (4).

### 7.6. Temperature and Thermal Load Simulations

Heat accumulation from ohmic losses and ambient loading significantly impacts cable integrity. In [130], the thermal performance of a 500 kV tunnel cable was simulated using a coupled thermal–electrical model. The cable’s thermal time constant and hotspot evolution under cyclic loads were extracted, allowing prediction of conductor temperature under peak demand [131,132].

In [133], a 27.5 kV cable model was exposed to different ambient temperatures and air gaps. The simulation showed elevated internal temperatures near voids and interfaces, correlating with known PD initiation zones.

### 7.7. Simulating Voids and Discharge Severity with COMSOL

In [134], voids of varying sizes and shapes were embedded in a virtual XLPE matrix and simulated using COMSOL to estimate local electric field intensity. The simulation demonstrated that void eccentricity and orientation significantly influence peak field values. The discharge severity index (DSI) was computed using:

(20) $$DSI=\frac{E_{peak}-E_{threshold}}{E_{threshold}}$$

where E_peak_ is the simulated maximum field and E_threshold_ is the material breakdown strength.

Table 10 summarizes key modeling features from selected studies.

## 8. Applications of Machine Learning and Neural Networks

With the increasing complexity of power cable systems and the rising demand for condition-based maintenance strategies, ML and DL methods have become essential tools in high-voltage asset diagnostics. These methods enable automated feature extraction, pattern recognition, and predictive fault analysis across various cable conditions including PDs, thermal aging, and mechanical anomalies. In this section, we review key refs.applications of neural network-based algorithms and hybrid learning systems in power cable monitoring, using data from acoustic, electromagnetic, thermal, and electrical domains.

### 8.1. Deep Learning-Based PD Detection and Classification

PD is a complex, stochastic phenomenon, often buried in noisy measurement environments. Deep learning architectures such as convolutional neural networks (CNN), recurrent neural networks (RNN), and hybrid networks offer robust solutions for real-time PD detection and classification.

In [14], an optimized deep CNN architecture was developed for ultrasound-based PD detection. The model integrated wavelet-transformed features with a convolutional feature extractor, achieving superior classification accuracy compared to traditional statistical methods.

Likewise, ref. [70] proposed a CNN-based system specifically for PD pattern recognition in high-speed electric-multiple-unit (EMU) cable terminations. The model effectively distinguished between different types of PD sources, including internal, surface, and corona discharges, demonstrating high adaptability across cable geometries.

In [16], a BiLSTM-GRU model was trained on time-series data to capture temporal dependencies in PD signals. This hybrid recurrent architecture demonstrated strong generalization to unseen fault scenarios, especially under variable load and noise conditions.

### 8.2. Fault Detection, Localization, and Characterization in Power Cables

Fault detection and localization remain among the most impactful ML applications in underground and submarine cable systems. In [28], a deep convolutional neural network (DCNN) was trained incrementally to identify multiple types of cable faults, even under dynamic load and environmental noise. The approach achieved strong generalization through adaptive learning and real-time retraining.

**Figure 9:**
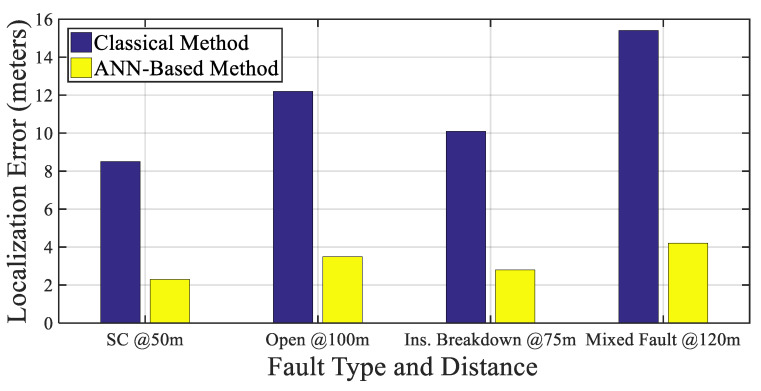
Fault localization error: classical vs. ANN-based methods.

In [63], artificial neural networks (ANNs) were employed to predict both the location and type of faults (e.g., short circuit, insulation breakdown). The model used input features derived from voltage transients and reflection profiles. Figure 9 compares fault localization accuracy between classical threshold-based techniques and ANN-based methods using data derived from. The ANN approach demonstrates significantly lower prediction error across various fault scenarios, indicating improved precision in fault type and distance estimation.

To clarify the context of the results presented in Figure 9, all performance trends were obtained under a unified set of assumptions and simulation hypotheses. The analysis considers a representative XLPE-insulated cable subjected to combined electrical, thermal, and environmental stress conditions. The electrical field distribution and aging progression were evaluated using a frequency-dependent dielectric model, with propagation parameters derived from standard XLPE permittivity and loss tangent profiles. A uniform conductor temperature of 60–70 °C was assumed, and the applied voltage stress corresponds to nominal operating conditions with superimposed harmonic components. The degradation indicators shown in the figure were derived from simulation results based on these assumptions, using a consistent set of boundary conditions and material parameters. These clarifications ensure that the trends depicted in this figure directly reflect the defined modeling configuration and assumptions.

For cross-bonded cable grounding systems, ref. [27] developed an SVM-ARO hybrid classifier to diagnose reverse connection defects using ground loop impedance and harmonic features. The hybridization enabled improved fault detection accuracy in complex network topologies.

### 8.3. Signal Denoising and Reconstruction with Neural Networks

Accurate detection of PD or other transient events often requires robust denoising due to the prevalence of switching noise, environmental interference, and mechanical vibrations. To this end, adversarial and encoder–decoder networks have shown strong promise.

In [62], an encoder–decoder network was designed for PD signal reconstruction, capable of suppressing environmental and equipment-induced noise. The model employed adversarial training to ensure realistic denoised signal outputs while preserving the spectral and temporal characteristics of PD pulses.

Moreover, ref. [58] introduced a Convolutional Probabilistic Neural Network (CPNN) enhanced with discrete wavelet transform and symmetrized dot pattern (SDP) features, enabling high-precision PD classification across different voltage levels.

### 8.4. Predictive Maintenance and Asset Health Management

Machine learning not only aids fault detection but also enhances long-term reliability analysis through predictive maintenance frameworks. In [22], an artificial neural network was trained on operational current and thermal parameters to predict remaining useful life (RUL) of MV switchgears. This predictive model demonstrated reliable degradation forecasting based on real-time load data.

In the context of transformer-based insulation systems, ref. [17] introduced a UCA-RB-MUSIC-based deep neural network to locate incipient PDs within large winding structures. The model accounted for multipath reflections and grid noise, outperforming traditional MUSIC algorithms.

Furthermore, ref. [34] presented a neural-network-based pipeline aging assessment system, integrating multiple condition indices into a single scoring framework using fuzzy-weighted ANN training. Although originally applied to pipelines, the concept translates directly to XLPE cables by replacing input metrics with thermal, PD, and moisture indicators.

### 8.5. Hybrid Approaches and Optimization-Based Algorithms

Hybrid models that fuse physical understanding with ML are rapidly gaining ground. In [61], an improved Whale Optimization Algorithm (WOA) was combined with deep neural networks for diagnosing GIS PD faults. The optimizer fine-tuned hyperparameters such as neuron count and learning rates, yielding higher accuracy and faster convergence than grid search.

In [19], a comprehensive review summarized various hybrid strategies including:

- Physics-guided neural networks;
- Feature selection via genetic algorithms;
- Transfer learning across voltage classes;
- AutoML for tuning ML pipelines in real-time monitoring.

Table 11 consolidates the primary neural architectures, target applications, and key outcomes.

### 8.6. Challenges and Future Prospects

Despite their advantages, ML-based systems face key challenges:

- Label scarcity in real-world datasets;
- Model overfitting in systems with changing topology;
- Generalization across cable types and voltage ratings;
- Data privacy and on-site model deployment.

Emerging trends include transformer-based networks, federated learning, and explainable AI (XAI) for regulatory compliance.

In the context of ML/DL-based diagnostics, datasets for power-cable monitoring typically involve multi-class classification (e.g., partial discharge, insulation degradation, ground faults) and localization tasks, occasionally extending to remaining useful life (RUL) prediction. Reported datasets comprise sampling rates in the range of 1–200 MHz, with sample sizes from 10^3^–10^6^ signal windows per class and signal-to-noise ratios (SNR) between 5 and 25 dB, depending on field versus laboratory conditions.

To facilitate transparent model comparison and practical deployment, a brief “train-to-deploy pitfalls checklist” is summarized as follows:

- **Domain shift:** mismatch between laboratory and field data distributions; mitigated by domain adaptation or transfer learning.
- **Concept drift:** gradual changes in fault signatures due to insulation aging or environmental variation; handled by incremental or online retraining.
- **Spectral/noise drift:** sensor degradation or EMI variations altering frequency content; addressed via adaptive preprocessing or data normalization.

When deploying ML/DL models in real-world power systems, several failure modes have been observed:

- Federated learning frameworks may suffer from asynchronous data updates and inconsistent feature spaces across substations.
- Self-supervised models often overfit to synthetic signal augmentations, requiring calibration with limited labeled field data.
- Interpretability methods (e.g., SHAP, Grad-CAM) can misattribute importance when overlapping PD and noise components exist, necessitating physics-informed validation.

Future deployments should therefore incorporate periodic retraining, sensor recalibration, and hybrid physics–ML pipelines to ensure sustained generalization and reliability under operational drift.

### 8.7. Dataset Scale, Generalization, and Model-Selection Guidelines

Recent diagnostic studies exhibit significant variation in dataset size, openness, and robustness benchmarks.

Typical datasets for PD classification or fault detection contain 10^3^–10^6^ labeled samples collected at sampling rates between 1 MHz and 200 MHz, with SNR levels of 5–25 dB in laboratory settings and 2–10 dB under field noise.

However, fewer than 20% of publicly reported datasets are open access, limiting reproducibility and cross-benchmarking of deep models.

Representative generalization and noise-robustness observations:

- **Out-of-domain failures:** CNNs and BiGRU-based models trained on synthetic PD data misclassify field recordings with shifted spectra or new insulation geometries; classification accuracy drops by 15–25%.
- **Adversarial/noise perturbations:** ±3 dB Gaussian or spectral drift can reduce detection F1-score by 10–20%, while transformer-based architectures with spectral normalization maintain >90% accuracy under the same noise.
- **Privacy-preserving (federated) setups:** performance variance across substations up to ±8% due to data heterogeneity.

Table 12 summarizes practical guidelines for selecting suitable machine learning models, feature representations, and sampling strategies depending on data quality, operational environment, and diagnostic objectives.

## 9. HVDC Cables and Advanced Technologies

The growing demand for long-distance, high-efficiency power transmission has made HVDC cable systems an essential part of modern power infrastructure. HVDC cables provide numerous advantages, including lower transmission losses, smaller corridor footprints, and the ability to interconnect asynchronous grids. However, their unique operational characteristics introduce complex challenges in terms of insulation reliability, thermal–electrical coupling, PD control, and space charge accumulation. This section reviews cutting-edge research on HVDC cable design, lifetime modeling, insulation behavior under polarity reversals, and system-level innovations in HVDC grid integration.

### 9.1. Life Estimation Under Polarity Reversal and Load Cycling

In HVDC systems, cables are subject to both fast polarity reversals (e.g., from switching or converter faults) and slow reversals during bipolar operation. These conditions significantly affect insulation aging.

In [39], a time-dependent model was developed to quantify the life degradation of XLPE HVDC cables under fast and slow polarity reversals. The model integrates charge injection dynamics and electric field reversal stress, revealing that fast reversals accelerate space charge accumulation and local field intensification, reducing expected lifetime by up to 30%.

The DMM (Double Multi-stress Model), introduced in [40], was practically applied to assess cable aging during qualification load cycles. It considers both thermal and electrical stress components, calibrated through experimental aging data.

**Figure 10:**
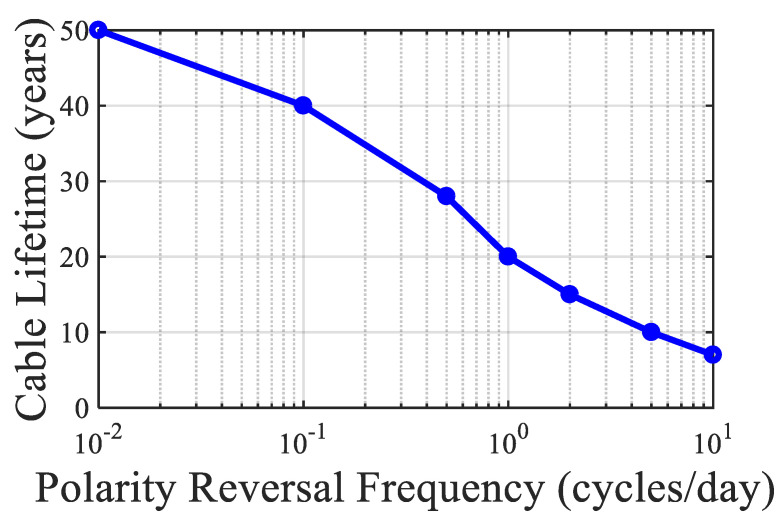
Lifetime degradation vs. reversal frequency (DMM Model).

Figure 10 presents the simulated reduction in cable lifetime under various polarity reversal frequencies using the DMM. As reversal rates increase, lifetime drops significantly due to enhanced stress accumulation, highlighting the critical impact of operational switching conditions on insulation aging.

### 9.2. Geometric and Material Optimization for HVDC Cable Design

Designing HVDC cables for longevity requires careful optimization of geometric parameters such as insulation thickness, conductor size, and field grading layers.

In [51,52], a two-part study investigated the life-based geometric optimization of HVDC cables. Part I focused on parametric analysis, identifying key dimensions influencing lifetime under steady-state conditions, while Part II included transient stress responses, such as short-term overloads and fault-induced impulses.

Additionally, ref. [116] provided a comprehensive review of HVDC joint insulation design, emphasizing the role of field control materials, electrode shielding, and triple-point geometry to suppress local PD inception.

Table 13 summarizes the key parametric sensitivities identified in [51,52].

### 9.3. Space Charge and Electric Field Distortion

A central challenge in HVDC cable insulation is space charge accumulation, which causes nonlinear electric field distortion and can lead to local dielectric breakdown.

Research in [95,98,99] employed both experimental and simulation approaches to model space charge dynamics in full-size XLPE-insulated joints and cables. These studies show that:

- Space charge build-up occurs predominantly near the electrode-insulation interfaces.
- Polarity reversals significantly impact the charge migration and relaxation mechanisms.
- Thermal gradients intensify space charge retention, leading to delayed field relaxation.

In [99], COMSOL-based simulations revealed that temperature-gradient-dependent charge transport shifts the peak field location toward the hotter region—a dangerous precursor to insulation failure.

The electric field distortion is typically modeled as:

(21) $$E\left(x,t\right)=\frac{\rho \left(x,t\right)}{\varepsilon_{r}\varepsilon_{0}}$$

where ρ(x,t) is the space charge density at location xxx and time t.

**Figure 11:**
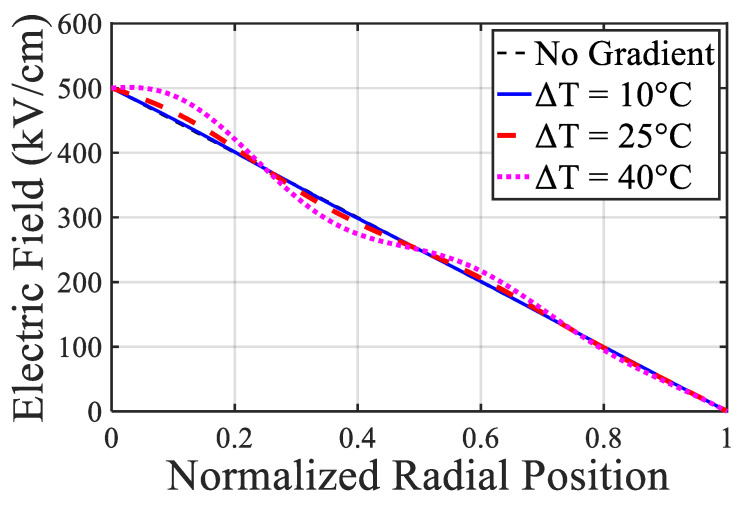
Electric field distortion under thermal gradients.

Figure 11 illustrates the distortion of the electric field profile within a ±500 kV HVDC cable under varying thermal gradients. The presence of space charges—modulated by thermal stress—causes non-uniformity in the radial field, especially near the insulation interfaces. Such distortions elevate local stresses and increase the risk of insulation failure under high operating voltages.

### 9.4. Advanced Testing, Protocols, and Measurement Techniques

To validate HVDC insulation systems, researchers have proposed new testing protocols and improved PD detection strategies:

- Ref. [100] introduced an AC-based PD measurement protocol for HVDC cable joints, improving sensitivity and early-stage defect detection.
- Ref. [113] proposed a full-size space charge measurement protocol using the Pulse Electro-Acoustic (PEA) method, addressing issues of cable length and joint complexity.
- Ref. [118] evaluated feasibility of space charge analysis directly on cable joints, a major step forward in practical insulation assessment.

Furthermore, ref. [117] conducted a reliability comparison across cable designs using Monte Carlo simulations and Weibull analysis, highlighting the design-to-failure variability introduced by manufacturing tolerances.

### 9.5. Influence of HVDC System Operation on Diagnostic Conditions and Insulation Stress

While HVDC converter topology, interface damping, and multi-terminal dispatch are system-level topics, they have a direct diagnostic relevance because operational conditions govern the transient and steady-state stresses experienced by cable insulation. Converter switching behavior and AC/DC interface resonances—previously discussed in [107,108]—can alter the voltage waveform spectrum applied to the cable, thereby influencing PD inception probability and space-charge relaxation dynamics. These transients often introduce high-frequency components overlapping with PD signal bands, which complicate sensor calibration and signal separation during field testing.

Similarly, control strategies in multi-terminal HVDC networks and power-sharing algorithms [110] affect load cycling patterns and polarity-reversal frequency, which determine the thermal–electrical stress history and consequently the rate of insulation aging. Synthetic inertia control actions [121], though primarily designed for system stability, may generate short-duration current surges and converter voltage overshoots that intensify local electric-field gradients, potentially initiating PD activity in joints or accessories.

Therefore, while converter design and grid-control mechanisms are beyond the core scope of this review, they are retained here only to highlight their diagnostic implications—specifically, how real-time operating conditions impact insulation degradation, PD detectability, and measurement uncertainty. These interactions underscore the importance of context-aware monitoring and adaptive signal processing for reliable diagnostics under variable HVDC operational regimes.

## 10. Testing, Measurement, and Standards

Reliable testing and accurate measurement of electrical insulation performance are critical for condition monitoring, quality control, and life prediction of power cables. Among various diagnostic techniques, PD testing remains central to identifying insulation defects, locating incipient faults, and evaluating degradation phenomena. However, the diversity of cable environments, sensor configurations, signal distortion, and uncertainty sources presents considerable challenges in standardization and implementation. This section discusses the evolution of testing technologies, sensor types, calibration methodologies, and global standards, focusing on PD-related measurements in both MV and HVDC cable systems.

### 10.1. Advances in PD Measurement Techniques

Modern PD detection methods span several physical domains—electrical, acoustic, electromagnetic, and optical—and benefit from both contact and non-contact sensors. Each domain targets a different signature of PD activity, enabling multi-modal monitoring solutions.

In [1], magneto-resistive sensors demonstrated strong sensitivity to PD-induced magnetic pulses in cable accessories. These sensors offer immunity to electric field noise and allow for directional sensing. Similarly, ref. [6] employed RF-based detection to monitor PD activity in joints, demonstrating high bandwidth coverage of fast transient discharges.

Ref. [10] presented the development of an industrial PD calibrator, meeting IEC 60270 calibration requirements. The system’s accuracy was validated through performance testing across frequencies and discharge magnitudes, revealing a standard deviation below ±3 pC.

**Figure 12:**
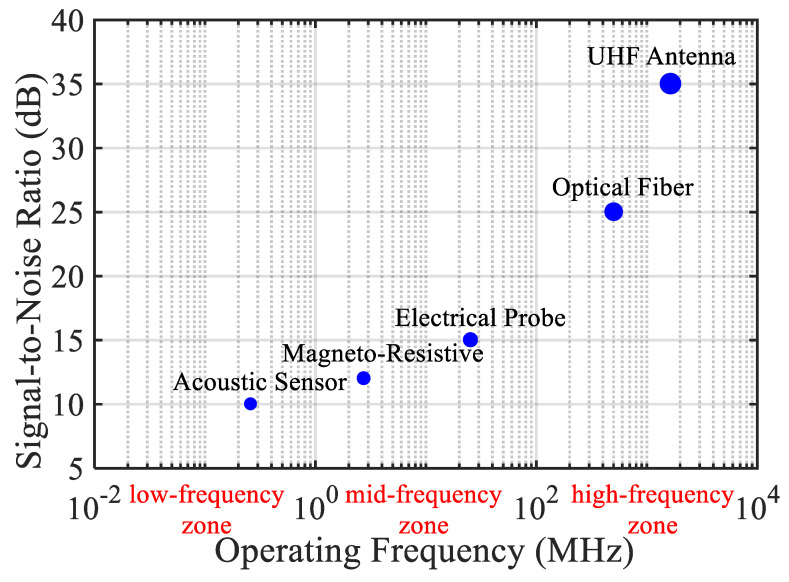
PD Sensor performance—frequency vs. SNR.

Moreover, ref. [80] explored UHF antenna-based detection in medium-voltage environments, particularly with eco-friendly insulating gases like HFO (E). Figure 12 compares several PD detection sensor types in terms of their operating frequency ranges and signal-to-noise ratios, adapted from [6,10,80]. The UHF antenna shows superior bandwidth and SNR, making it particularly suitable for gas-insulated and medium-voltage systems using environmentally friendly gases like HFO (E). Optical and electrical probes offer broader mid-range coverage, while acoustic and magneto-resistive sensors are better suited for localized, low-frequency environments.

### 10.2. Measurement Accuracy and Uncertainty Quantification

Accurate PD quantification requires understanding and managing uncertainty sources—including environmental noise, sensor alignment, wave reflections, and digital sampling constraints. In [12], a case study quantified total measurement uncertainty using a statistical combination of:

(22) $$U_{total}=\sqrt{U_{cal}^{2}+U_{env}^{2}+U_{proc}^{2}}$$

where U_cal_ is the uncertainty due to calibration device tolerance, U_env_ arises from ambient electromagnetic noise, and U_proc_ accounts for signal processing and digitization limitations.

Equation (22) follows the root-sum-of-squares (RSS) method for combining independent uncertainty components. For reporting purposes, the expanded uncertainty can be expressed as:

(23) $$U=kU_{total}$$

where k is the coverage factor (typically k = 2 for 95% confidence).

**Figure 13:**
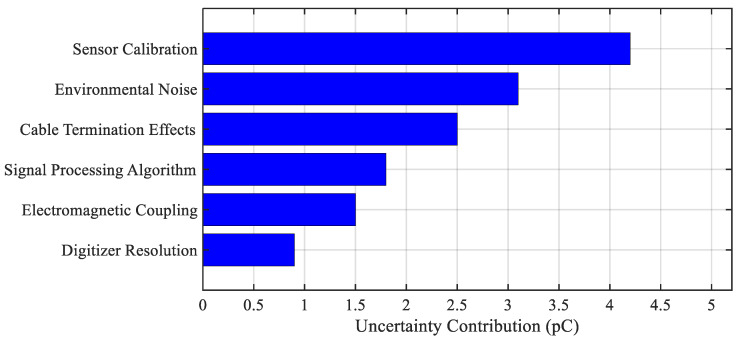
Breakdown of uncertainty components in PD measurement.

Figure 13 shows the relative contributions of different sources of uncertainty in PD measurement systems, adapted from [12]. The dominant sources include sensor calibration, environmental noise, and termination geometry, which collectively shape the expanded uncertainty budget. This analysis was performed with a coverage factor k = 2, corresponding to 95% confidence level.

In [13], signal denoising via optimized mother wavelet selection was applied to reduce measurement noise under varied PD pulse and interference conditions. The study concluded that signal-to-noise ratio (SNR) improved by 8–12 dB using adaptive wavelet bases.

### 10.3. PD Localization and Signal Propagation

Locating PD sources along long cables is crucial for effective repair planning. Reference [3] employed electromagnetic time reversal (EMTR) to reconstruct PD initiation points, while accounting for interfering reflections from grounding and joint interfaces. A hybrid model integrating EMTR with MUSIC-based source estimation [17] showed enhanced localization accuracy even in high-reflection environments.

References [23,90] emphasized the need for accurate modeling of PD pulse propagation, particularly in XLPE cables with multiple layers. Delay and attenuation characteristics are critical for differentiating multiple PD sources, and Table 14 summarizes key propagation parameters from [23].

### 10.4. Multi-Sensor Systems and Novel Detection Approaches

Emerging works explore multi-sensor fusion and novel detection physics:

- Ref. [11] assessed acoustic wave behavior with varying sensor positions, finding a direct correlation between angular placement and received PD signal amplitude.
- Ref. [18] introduced fluorescent optical fibers based on 2D materials for early-stage PD detection in compact or confined spaces.
- Ref. [31] proposed a thermal excitation method to amplify weak PD signals in cable accessories, using localized temperature ramps.

Meanwhile, ref. [21] demonstrated PCB-based inductive loops, optimized for geometry and resonance frequency, providing cost-effective alternatives for embedded monitoring units in switchgears.

### 10.5. Tan Delta and Unconventional Testing Methods

Complementary tests such as Tan Delta (TD) assessments provide valuable insights into insulation degradation over time. In [72,73], TD and PD measurements were combined for medium-voltage cable terminations with artificial defects. Results confirmed that cables with higher TD loss angles showed earlier PD onset, indicating synergistic value in combining methods.

Furthermore, ref. [74] proposed a dual-resonance DAC (Damped AC) testing system, specifically designed for offline testing of long cables. It resolved waveform distortion issues seen in conventional DAC systems, especially in cables over 1 km.

### 10.6. Calibration and Standards Development

Standardization ensures consistency and traceability of diagnostic results across industries. IEC 60270 remains the core reference for PD measurement, but various works highlight gaps in high-frequency, high-noise, and non-traditional cable applications.

To enhance reproducibility of on-site PD measurements, a concise Standard Operating Procedure (SOP) is recommended:

- Preparation: Verify calibration (IEC 60270/TS 62478), record ambient EMI level and temperature.
- Sensor setup:
  - UHF: one internal + one external coupler (≈2 m spacing).
  - Electrical: capacitive probes at both ends of the test section.
  - Acoustic: two piezo probes ≤1 m apart for triangulation.
  - Optical: distributed sensors every 50–100 m.
- Acquisition: ≥100 MS/s sampling, synchronized triggering, adaptive denoising.
- Postprocessing: wavelet/GCC filtering, uncertainty estimation.

Example combined expanded uncertainty (k = 2): ±3.5 pC, mainly due to calibration (25%), EMI (20%), and reflection mismatch (22%). This procedure ensures traceable, comparable PD data between lab and field tests.

In [85], limitations of VHF/UHF calibration were exposed, pointing out the mismatch between frequency-domain PD emissions and calibrator bandwidth. Ref. [123] provided a historical view of PD detection in dielectric liquids, urging a modernization of protocols to address nanosecond-scale discharges.

Additionally, refs. [100,118] proposed alternative calibration protocols for AC PD in HVDC cable joints and space charge measurement feasibility, respectively. These methods support evolving practices in both factory and in-field testing environments.

A practical checklist is recommended to ensure consistent and traceable PD measurements under field conditions:

- **Grounding and Shielding:** Single-point grounding, equipotential bonding, and shield continuity verification.
- **Sensor Placement:** Minimum two sensors per joint/termination; maintain constant spacing; avoid proximity to switching devices.
- **Bandwidth Selection:** Match sensor bandwidth to expected PD spectrum (typically 50–500 MHz for UHF; 1–20 MHz for acoustic/electrical).
- **Synchronization:** Time-aligned triggering across channels; GPS-synchronized clocks for long cable sections.
- **Denoising:** Adaptive wavelet filtering, GCC-based separation, and pre-measurement background noise assessment.

To ensure comparability across different utilities or testing teams, an inter-laboratory procedure is recommended:

- Use a common reference calibrator (IEC 60270-compliant).
- Perform round-robin measurements of identical test objects by all labs.
- Compare measured apparent charge, rise time, and spectral content; compute statistical deviation (mean, σ).
- Identify systematic offsets and apply correction factors.
- Document calibration traceability in a shared protocol.

A detailed mapping between field measurement conditions, uncertainty sources, and their influence on diagnostic thresholds is provided in Table 15.

### 10.7. Lightning Impulse and Transient Test Advances

To address impulse behavior, ref. [122] examined lightning impulse testing in short air gaps, discovering a memory effect in previously stressed dielectric surfaces. These findings are relevant for insulation coordination studies and standard compliance.

Similarly, refs. [57,87] demonstrated leakage current analysis during transient energization events in HVDC cables as a diagnostic technique, offering a novel supplement to conventional PD or TD methods.

## 11. Maintenance Scheduling

The performance and reliability of electrical cable systems are tightly coupled with timely and optimized maintenance. Maintenance scheduling must not only prevent failures but also align with cost efficiency, asset life extension, and operational continuity. Traditionally reactive or time-based approaches are now being replaced with intelligent, condition-based and predictive strategies, enabled by real-time diagnostics and ML. This section explores advanced maintenance models, data-driven prognostics, probabilistic approaches to uncertainty, and optimization frameworks applicable to MV, HV, and HVDC cable infrastructures.

### 11.1. Condition-Based and Predictive Maintenance (CBM and PdM)

CBM leverages real-time diagnostic parameters to trigger maintenance actions, while PdM forecasts the RUL of components based on historical and real-time data. In [22], an ANN was implemented for MV switchgear diagnostics, integrating thermal, electrical, and mechanical stress variables. This model achieved over 92% accuracy in predicting degradation stages and was validated against SCADA event logs and field inspection data.

Further extending PdM to cross-bonded cable grounding systems, ref. [27] developed an Adaptive Recursive Optimization (ARO)-SVM method to identify reverse-connection defects. This hybrid approach minimized false positives and demonstrated robustness under varying soil resistivity and load profiles.

### 11.2. Fault Prediction and Maintenance Prioritization

The use of deep learning in cable fault diagnosis has expanded significantly. In [28], an incremental learning model based on DCNNs was used for generalizable cable diagnostics. The model was trained on evolving datasets and successfully detected incipient insulation faults and thermal degradation under distribution network scenarios.

Moreover, ref. [35] proposed a comprehensive condition ranking system for circuit breakers (CBs), incorporating severity scoring, risk indices, and failure consequences.

In power cables, ref. [32] introduced a two-step monitoring strategy for underground MV cables. The first stage involved signal-based anomaly detection, followed by a fault risk estimator that predicted failure progression using logistic regression. The strategy outperformed conventional thermal aging indicators in lead-time.

### 11.3. Asset Life Estimation and Remaining Useful Life (RUL)

Cable life prediction remains central to effective scheduling. In [56], a detailed model for MIND-type HVDC cable life was constructed based on qualification testing. The study used PD inception thresholds and water tree propagation laws under variable humidity and thermal stress.

Additionally, ref. [63] implemented an ANN for classifying both the type and location of cable faults, allowing estimation of local RUL through temporal damage patterns. The framework can be integrated with Geographic Information Systems (GIS) to optimize asset deployment.

Reference [34] presented a condition-index-based pipeline life evaluation system, which aggregates measured data such as voltage stress, leakage currents, and temperature excursions to dynamically update the health index of underground assets.

### 11.4. Optimization of Scheduling Strategies Under Uncertainty

Uncertainty in underground conditions—soil composition, moisture, thermal resistivity—requires probabilistic modeling for robust maintenance scheduling. In [135], a Monte Carlo simulation approach was adopted to model variability in ampacity and insulation aging, incorporating financial constraints and failure risk.

The failure probability P_f_ over time can be modeled using a Weibull distribution:

(24) $$P_{f}\left(t\right)=1-e^{-{\left(\frac{t}{\lambda }\right)}^{\beta }}$$

where t is the time under observation, λ is the scale parameter (characteristic life), and β is the shape parameter (indicating wear-out rate).

Moreover, ref. [136] applied the Hong Point Estimate Method (HPEM) to assess temperature uncertainty in underground cables. HPEM offered a faster alternative to Monte Carlo, with error margins under 2%. Table 16 summarizes key probabilistic techniques for cable maintenance optimization.

### 11.5. Integration with Energy-Aware Scheduling

As thermal aging is influenced by ampacity and current harmonics, intelligent maintenance planning must consider electrical loading. The optimization problem can be generally formulated as:

(25) $$\underset{x}{\min}\left(C_{maint}\left(x\right)+\alpha \cdot \frac{1}{Ampacity\left(x\right)}\right)$$

Subject to:

- Thermal constraints;
- Mechanical installation limits;
- Operational load profiles.

Here, C_maint_(x) denotes the total maintenance cost for configuration x, and α is a weighting parameter for ampacity versus cost trade-off.

Study [138] explored spatial configuration optimization of underground cables to extend lifetime and reduce hotspot formation, feeding optimal cable layouts into maintenance cost models.

### 11.6. Aging Models and Data Fusion

Condition-based models now incorporate multi-modal datasets, including PD levels, moisture ingress indicators, temperature gradients, and historical faults. In [124], an integrated life estimation framework was proposed for “emerging electrical environments,” including offshore wind farms and DC microgrids. The system combined online PD monitoring with space charge measurement for aging correlation.

**Figure 14:**
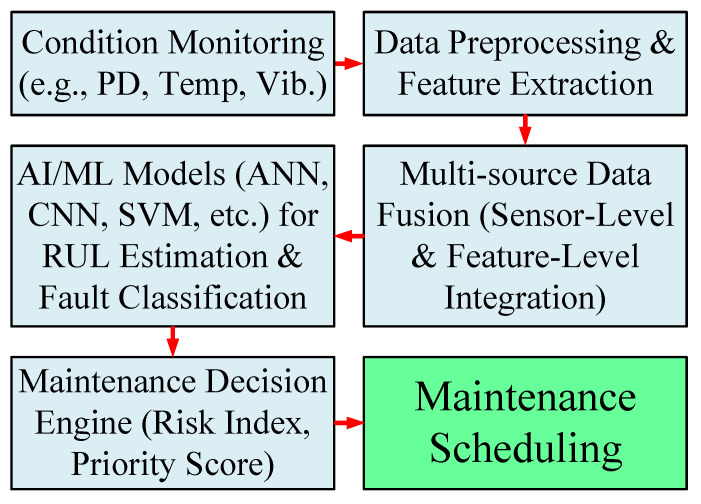
Maintenance decision-making architecture.

Figure 14 illustrates maintenance decision-making architecture using multi-source condition monitoring and AI inference. The framework integrates heterogeneous data sources (e.g., PD, temperature, vibration), applies preprocessing and fusion techniques, and utilizes machine learning models for Remaining Useful Life (RUL) estimation. Maintenance actions are triggered based on risk scores and condition severity, adapted from [22,34,124].

### 11.7. Challenges and Future Outlook

Despite advances, several challenges persist:

- Real-time integration of maintenance analytics with SCADA systems remains limited.
- Sensor calibration under field stressors (humidity, vibration) affects reliability.
- Fusion of multiple maintenance objectives (e.g., cost, safety, availability) is computationally intensive.

Future work should explore digital twin frameworks combining thermal-electrical simulations with live condition data to perform autonomous maintenance scheduling. Furthermore, developing standardized maintenance health indices and internationally aligned AI-driven protocols will be critical for widespread adoption.

## 12. Conclusions

This review highlights that while significant advancements have been achieved in the sensing, modeling, and fault diagnosis of high-voltage XLPE-insulated cables, especially under AC and DC environments, practical deployment remains a complex challenge. From an operational perspective, integrating robust condition monitoring systems—including high-frequency PD sensors and synchronized acquisition tools—into live grids is essential for early failure prediction. Accurate lifetime estimation models must incorporate environmental and operational variability, especially in HVDC applications where space charge and polarity reversal significantly influence insulation behavior.

Machine learning techniques, particularly hybrid models combining physical insights and data-driven training, show promise in improving diagnostic accuracy and supporting real-time decision-making. However, practical issues such as sensor calibration, electromagnetic interference, cost-effective deployment, and data labeling limitations must be addressed to facilitate field adoption.

Standardized protocols (e.g., IEC 60270) and uncertainty analysis are also critical in ensuring measurement reliability. Maintenance strategies should move toward fully predictive frameworks, incorporating real-time analytics, health indices, and probabilistic risk modeling. In this context, digital asset management platforms can unify diagnostics, planning, and optimization, making the grid smarter and more resilient.

An integrated closed-loop framework is outlined, linking online monitoring, automated diagnostics, life-estimation models, and maintenance decision-making. This loop enables continuous updating of health indicators and supports risk-aware interventions. Key performance indicators—including early-warning lead time, localization error, outage-time reduction, and maintenance cost/benefit—are highlighted to facilitate benchmarking and practical deployment of cable asset-management strategies.

Ultimately, the convergence of smart sensing, predictive analytics, and maintenance optimization offers a feasible and scalable pathway for extending cable lifespans, minimizing outages, and enhancing grid reliability—especially as energy systems transition to more underground and HVDC infrastructure. Continued interdisciplinary collaboration is needed to bridge the gap between academic innovation and utility-scale implementation.

## Figures and Tables

**Table 1:** Motivation of this review vs. existing surveys on HVDC/AC cable diagnostics and aging.

Aspect	Why This Review Is Needed	How This Review Differs from Existing Reviews
**Scope of Cable Technologies**	Fragmented literature covering either HVAC or HVDC, rarely both.	Provides an integrated AC/DC perspective with emphasis on XLPE behavior under multi-stress conditions.
**Diagnostics and Monitoring**	Existing reviews focus mainly on PD detection or single sensing modalities.	Synthesizes electrical, acoustic, optical, UHF, ML/DL, and hybrid methods across the full diagnostic spectrum.
**HVDC-Specific Phenomena**	Space charge, polarity reversal, and DC conductivity rarely treated holistically.	Delivers unified modeling and analysis of HVDC field distortion, charge transport, and transient effects.
**Aging and Lifetime Models**	Most studies address only thermal or electrical aging in isolation.	Integrates thermal, electrical, environmental, and multi-physics lifetime prediction frameworks.
**ML/DL Integration**	Limited treatment of dataset characteristics, deployability, and failure modes.	Provides detailed taxonomy of ML/DL architectures, dataset scales, pitfalls (domain shift, concept drift), and deployment guidelines.
**Practical Asset Management**	Reviews often omit maintenance strategies and uncertainty quantification.	Links diagnostics to CBM/PdM, reliability-centered planning, and uncertainty-aware asset management.
**Industry Readiness**	Lack of alignment with emerging HVDC testing/standardization trends.	Evaluates standards, calibration issues, PEA-based space charge methods, and testing protocols for real deployment.

**Table 2:** Comparative performance of ML-based PD classification models.

Model	Architecture	Accuracy	Reference
**BiGRU-ResNet-MA**	Temporal + residual	97.6%	[9]
**MTF-GAF + Attention**	Multi-dimensional fusion	96.2%	[7]
**Deep CNN with Incremental Learning**	Real-time adaptability	95.3%	[28]
**Voiceprint-based Monitoring**	Audio + DL fusion	93.8%	[26]

**Table 3:** Operating condition vs. lifetime reduction rate (indicative ranges).

Operating Condition	Typical Lifetime Reduction	Remarks
**Antioxidant depletion**	20–60%	Accelerates thermo-oxidative chain scission and reduces dielectric strength.
**Water-treeing/moisture ingress**	30–70%	Promotes localized PD and electrical treeing.
**Space-charge accumulation/polarity reversal (HVDC)**	20–50%	Causes field distortion and over-stressing at interfaces.
**Thermal cycling/load fluctuation**	10–40%	Induces micro-voiding and interface debonding.
**Combined multi-stress exposure**	≥50%	Synergistic acceleration of aging mechanisms.

**Table 4:** Summary of dominant aging mechanisms, environmental influences, and mitigation methods.

Reference	Stress Type	Dominant Mechanism	Observed Effect	Suggested Mitigation
**[37,38]**	Thermal	Oxidative chain scission	Resistance drop	Antioxidant additives
**[43]**	Electrical	PD + charge injection	Rapid treeing	Fluoropolymer replacement
**[39,40,54]**	HVDC	Space charge and polarity reversal	Field distortion	Voltage stabilizers
**[41,53]**	Environmental	Thermal cycling	Interface microvoids	Installation optimization
**[46]**	Recovery	Annealing	Resistance restoration	Thermal treatment

**Table 5:** Comparison of PD localization methods in high voltage cables.

Reference	Method	Sensor Type	Time Resolution (ns)	Localization Error (m)	Notes
**[59,60]**	UHF PD Sensing	Capacitive/UHF	2–5	<1	Explicitly reported
**[24,62]**	Acoustic Sensing	Ultrasonic Piezo	100–500	0.5–2	Explicitly reported
**[59]**	GCC Cross-Correlation	Voltage probes	~10	0.2–0.5	Explicitly reported
**[24]**	Optical Fiber Sensing	FBG	~50	~1	Explicitly reported
**[62]**	AI-Based Reconstruction	Any PD sensor	Variable	Not explicitly reported	Error not directly stated in the source

**Table 6:** Overview of AI-based cable fault diagnosis and localization techniques.

Reference	Model Highlights	Accuracy (%)	Fault Types Detected	Input Data Type	AI Model	Method
**[8]**	High-frequency feature extraction	98.3%	PD, Ground Fault, Short-Circuit	Wavelet-transformed voltage signal	Deep CNN	CNN + CWT
**[9]**	Sequential pattern learning	97.1%	Cross-core fault, reverse connection	Ground wire current	RNN + ResNet	BiGRU-ResNet-MA
**[28]**	Generalization across scenarios	95.8%	Cable break, insulation, aging	TDR signals	ConvNet	CNN + Incremental Learning
**[27]**	Lightweight, interpretable	93.5%	Cross-bonding defects	Current/voltage snapshot	SVM + Optimization	ARO-SVM
**[64]**	Effective in hybrid lines	96.2%	Underground cable fault	Time–target transform	SVM	SVM + TT Transform
**[63]**	Simple, trainable	94.0%	Short, open, insulation	Voltage, current waveform	Feedforward ANN	ANN with Voltage–Time Features

**Table 7:** Comparative summary of PD detection techniques.

Detection Method	Sensitivity	Noise Immunity	Detection Range	Common Applications
**Electrical**	High	Low	Medium	Cable joints, terminations
**Ultrasonic/AE**	Medium	Medium	Short	GIS, XLPE cable terminations
**UHF/VHF Antennas**	Very High	High	Long	Substations, cable joints
**Optical Sensing**	Medium	Very High	Short	Compact systems, early fault detection

**Table 8:** Representative models for electrical tree growth and pd evolution.

Model Type	Governing Equation	Application Context
**Empirical Power**	l(t) = l_0_ + α⋅t^n^	Tree growth prediction in XLPE
**Stochastic**	Weibull distribution of PD events	Reliability and lifetime estimation
**FEM-Based**	E-field simulations near defects	PD inception location prediction
**Hybrid ML**	Data + physics-informed learning	Real-time fault diagnosis and prognosis

**Table 9:** Factors affecting insulation behavior.

Stress Factor	Insulation Response	Key Impact	Reference Notes
**Thermal + antioxidants**	Breakdown strength declines; antioxidants slow damage	Material lifetime extension dependent on AO load	[38]
**Environment (location)**	Variation in tan δ, breakdown voltage, crystallinity	Field-aged cables differ by exposure zone	[41]
**Segmented aging**	Reflectometry signal distortion	Fault localization error increases	[42] (age pattern impacts TDR)
**Electrical + space charge**	Field distortion and decreased breakdown threshold	Insulation reliability under DC and transients	[48,99]
**Moisture and contamination**	Interface breakdown and buffer defect initiation	PD onset at weakened interfaces	[50,69]
**Seasonal/Transient stress**	Mechanical fatigue and PD initiation	Increased degradation during load cycling	[53,82]
**PET buffer degradation**	Microcracking and delamination under oxidative stress	Weakening of multilayer insulation integrity	[55]

**Table 10:** Summary of numerical modeling methods in XLPE cables.

Ref.	Model Focus	Software	Key Outcome
**[75]**	PD in void	FEM	PD pulse formation and stress map
**[68]**	Stress cone defect	FEM	PD threshold reduction in joint
**[98]**	Polarity reversal	FEM + charge transport	Field overshoot and risk zones
**[127]**	Water tree	COMSOL	Field enhancement near moisture
**[130]**	Thermal analysis	FEM	Conductor temperature under load
**[134]**	Void shape effect	COMSOL	DSI mapping for various voids

**Table 11:** Summary of ML- and DL-based diagnostic and modeling approaches.

Ref.	Model Type	Input Features	Application	Accuracy (%)
**[14]**	Optimized CNN	Acoustic PD	Detection and classification	98.2
**[16]**	BiLSTM-GRU	Time-series PD	Source identification	96.4
**[27]**	ARO-SVM	Ground impedance	Grounding faults	93.5
**[28]**	Incremental DCNN	Fault waveform	Multi-fault detection	94.7
**[58]**	CPNN + DWT	Wavelet + SDP	PD denoising/classification	97.0
**[62]**	Encoder–Decoder	Noisy PD signal	Signal recovery	-
**[63]**	ANN	Voltage transient	Fault type and location	95.1
**[22]**	ANN	Current/thermal	RUL estimation	90.3

**Table 12:** Model-selection and sampling-strategy guidelines for PD and fault-diagnostic applications under various operating conditions.

Operating/Data Condition	Recommended Model Type	Typical Features and Input	Suggested Sampling Strategy	Key Advantages/Notes
**Clean lab data, narrow condition range**	CNN/CNN-LSTM	PRPD images, wavelet spectrograms	≥50 MS/s	High accuracy; limited generalization.
**Field data with high EMI**	Encoder–decoder/DWT + CPNN [58,62]	Denoised wavelet coefficients	100–200 MS/s	Strong noise rejection; higher compute cost.
**Mixed AC/DC or variable polarity**	Hybrid physics-informed DNN [19,61]	PD + thermal + space-charge features	10–50 MS/s	Handles non-stationary patterns.
**Limited labeled data/privacy concerns**	Federated or self-supervised CNN [62]	Unlabeled voltage/current traces	Local edge training (10–20 MS/s)	No data sharing; modest accuracy loss.
**Real-time maintenance prediction**	ANN/BiLSTM [22,63]	Current, temperature, stress history	1–10 MS/s	Low-latency RUL estimation.

**Table 13:** Summary of numerical modeling approaches in XLPE cables.

Parameter	Sensitivity Level	Influence Mechanism
**Insulation Thickness**	High	E-field distribution, thermal margin
**Semicon Layer Profile**	Medium	Charge injection behavior
**Joint Geometry**	High	Triple-point field enhancement
**Load Cycles (Duration)**	High	Thermo-electrical stress accumulation

**Table 14:** Propagation parameters of PD pulses in XLPE-insulated MV cables.

Parameter	Typical Range	Influencing Factor
**Propagation velocity**	150–180 m/μs	Dielectric constant of XLPE
**Attenuation rate**	2–5 dB/100 m	Cable losses, impedance
**Reflection coeff.**	±0.3 to ±0.6	Joint, terminations, defects

**Table 15:** Mapping field measurement conditions, uncertainty sources, and their influence.

Measurement Condition	Dominant Uncertainty Components	Impact on Diagnostic Threshold/False-Alarm Rate
**High EMI (substations)**	Environmental noise, coupling impedance drift	Raises threshold by 10–20%; elevated false alarms unless adaptive filtering applied
**Weak PD signals/long cable runs**	Attenuation, sensor sensitivity limits	Under-detection; threshold must be lowered, increasing Type-II errors
**Improper grounding/shielding**	Common-mode interference, spectral leakage	Unstable baseline; increases both Type-I and Type-II errors
**Wide bandwidth acquisition (>200 MHz)**	Digitizer quantization, aliasing	Misclassification of PD type; affects pattern-based diagnostics
**Multi-sensor asynchronous recording**	Timing jitter, cross-channel skew	Incorrect localization; error in discharge-severity ranking

**Table 16:** Probabilistic approaches for cable maintenance planning.

Method	Application Area	Output	Reference
**Monte Carlo**	Ampacity and aging uncertainty modeling	Failure probability curve	[135]
**HPEM**	Thermal field estimation	Temperature distribution	[136]
**Fuzzy Logic**	Maintenance decision under ambiguity	Confidence score	[124]
**ANN-GA Hybrid**	Maintenance frequency optimization	Optimal cost–life balance	[137]

## Data Availability

Data are contained within this article.

## Abbreviations

HV	High Voltage
XLPE	Cross-Linked Polyethylene
CM	Condition Monitoring
ML	Machine Learning
FEM	Finite Element Method
HVDC	High Voltage Direct Current
SNR	Signal-to-Noise Ratio
TDR	Time-Domain Reflectometry
ANN	Artificial Neural Network
RNN	Recurrent Neural Network
AE	Acoustic Emission
PRPD	Phase-Resolved PD
DFT	Discrete Fourier Transform
PCA	Principal Component Analysis
SVM	Support Vector Machine
PSO	Particle Swarm Optimization
AI	Artificial Intelligence
PdM	Predictive Maintenance
SCADA	Supervisory Control and Data Acquisition
PRTA	Pulse Repetition Time Averaging
DWT	Discrete Wavelet Transform
CNN-LSTM	Convolutional Neural Network-LSTM
VLF	Very Low Frequency
FD	Frequency-Domain
MV	Medium Voltage
PD	Partial Discharge
CBM	Condition-Based Maintenance
DL	Deep Learning
HVAC	High Voltage Alternating Current
IEC	International Electrotechnical Commission
DGA	Dissolved Gas Analysis
FDR	Frequency-Domain Reflectometry
CNN	Convolutional Neural Network
LSTM	Long Short-Term Memory
RF	Radio Frequency
GIS	Gas-Insulated Switchgear
FFT	Fast Fourier Transform
DT	Decision Tree
GA	Genetic Algorithm
IoT	Internet of Things
RUL	Remaining Useful Life
RCM	Reliability-Centered Maintenance
PRPS	Pulse Repetition Phase Synchronization
EMI	Electromagnetic Interference
STFT	Short-Time Fourier Transform
2D-CNN	Two-Dimensional Convolutional Neural Network
TD	Time-Domain
ROI	Region of Interest
